# Examination of China’s performance and thematic evolution in quantum cryptography research using quantitative and computational techniques

**DOI:** 10.1371/journal.pone.0190646

**Published:** 2018-01-31

**Authors:** Nicholas V. Olijnyk

**Affiliations:** United States Military Academy, West Point, NY, United States of America; Bar-Ilan University, ISRAEL

## Abstract

This study performed two phases of analysis to shed light on the performance and thematic evolution of China’s quantum cryptography (QC) research. First, large-scale research publication metadata derived from QC research published from 2001–2017 was used to examine the research performance of China relative to that of global peers using established quantitative and qualitative measures. Second, this study identified the thematic evolution of China’s QC research using co-word cluster network analysis, a computational science mapping technique. The results from the first phase indicate that over the past 17 years, China’s performance has evolved dramatically, placing it in a leading position. Among the most significant findings is the exponential rate at which all of China’s performance indicators (i.e., Publication Frequency, citation score, H-index) are growing. China’s H-index (a normalized indicator) has surpassed all other countries’ over the last several years. The second phase of analysis shows how China’s main research focus has shifted among several QC themes, including quantum-key-distribution, photon-optical communication, network protocols, and quantum entanglement with an emphasis on applied research. Several themes were observed across time periods (e.g., photons, quantum-key-distribution, secret-messages, quantum-optics, quantum-signatures); some themes disappeared over time (e.g., computer-networks, attack-strategies, bell-state, polarization-state), while others emerged more recently (e.g., quantum-entanglement, decoy-state, unitary-operation). Findings from the first phase of analysis provide empirical evidence that China has emerged as the global driving force in QC. Considering China is the premier driving force in global QC research, findings from the second phase of analysis provide an understanding of China’s QC research themes, which can provide clarity into how QC technologies might take shape. QC and science and technology policy researchers can also use these findings to trace previous research directions and plan future lines of research.

## Introduction

National governments have shown considerable interest in quantum cryptography (QC) for national security purposes with policy researchers highlighting the People’s Republic of China (China) as the most ambitious and the United States (US) as falling behind [1,2]. China has placed significant emphasis on science and technology (S&T) research in QC for over two decades [2]. According to Hurst, research into quantum computing began in the 1990s at China’s National University of Defense Technology and shortly thereafter by the People’s Liberation Army’s University of Science and Technology [2]. The Key Laboratory of Quantum Information at the University of Science and Technology of China was developed in 2001 to take a lead role in quantum computing research. Over the next several years, Chinese experts projected confidence in their ability to develop quantum computing technology. However, Hurst identified a potential shift in China’s research focus, which moved from the more difficult road of attempting to develop quantum computing machines to the more practical option of developing QC systems. Then, China exceeded a previously held world record in 2013 with the development of a quantum gate operation on an electron far faster than had previously been accomplished [2]. China has reasserted its vision of engineering several quantum computer components (e.g., quantum computer chips, quantum routers, and quantum networks) and claims to have developed the first quantum cryptographic system to secure a government network [2]. Reports have indicated that China launched the first satellite dedicated to experimenting with quantum communication systems in 2016 [3]. China has made many advancements in quantum computing over the years and still remains dedicated as shown by its growing investment [1].

Furthermore, QC can be used to create communication systems that are “unhackable,” thereby “fundamentally alter[ing] the strategic balance and stability of the cyber domain” [1,2,4,5]. However, policy researchers argue that “the future trajectory of these technologies remains uncertain” [1]. The current study alleviates some of the aforementioned “uncertainty” by bridging the research policy and scientometrics research specialties to provide a quantitative examination of China’s QC research.

The following two research questions (RQs) guided this investigation: 1) how is China’s international performance with regard to QC research evolving compared with that of other countries and 2) how have China’s main QC themes evolved?

### Background

The following section provides a brief description of scientometrics and QC for those readers not familiar with these domains.

#### Scientometrics

The creation and use of large-scale S&T research metadata [6] in conjunction with quantitative and computational techniques has led to a robust research field known as scientometrics [7]. Scientometrics has a host of established techniques for objectively evaluating S&T research performance and visually mapping the evolution of research themes [8]. One main aim of applied scientometric research is to reduce complex large-scale S&T activity to manageable terms, thereby decreasing the cognitive load for national S&T managers, decision-makers, and scientists [9–11].

Scientometrics is a well-established research method that mainly analyzes the quantifiable characteristics of metadata derived from S&T research documents to determine research performance, intellectual structure, and evolving research patterns [12]. Moreover, scientometrics applies mathematical techniques (e.g., statistical models, network analysis, etc.) to the quantitative and qualitative inputs and outputs of scientific units (keywords, documents, authors, institutions, and countries) to generate useful indicators [10]. S&T research funding can be considered an input and research publications and citations can be considered an output. Although not included in the current study, patents are another output for measuring S&T research. Citation scores and publication frequency are often considered to operationalize research quality and quantity, respectively [13]. A citation, which is synonymous with a reference, is a symbolic token used for acknowledgment or to draw a semantic connection between documents. Co-occurrence analyses (using words, citations, or authors) allow for the creation of maps that depict the S&T research theme structure and plotting structure over time can provide a view of research evolution, with historically important topics or emerging research fronts frequently depicted [13].

Scientometrics is closely associated with bibliometrics, which is a quantitative (often statistical) analysis method used to study publication patterns. Scientometrics overlaps with bibliometrics when S&T research activity is replaced by S&T communication, which often occurs through formal channels (e.g., S&T publications). Scientometrics uses bibliometric data (i.e., quantifiable publication metadata) and other data types to investigate the structure and behavior of S&T research. However, bibliometrics does not need to focus on S&T publications, and scientometrics does not have to use bibliometric data.

#### Quantum cryptography

The other technical research domain addressed in this study combines quantum mechanics with cryptography. Cryptography is the science and practice of maintaining the privacy and confidentially of information by applying systematic obfuscation. A cryptographic system takes information as input (plain text) and then computationally processes the information using a special algorithm (cipher) along with a set of secret parameters (cipher key), thereby transforming (or encrypting) the information into a meaningless form (cipher text). The process should be able to be reversed using only the cipher key, which decrypts the cipher text back into a meaningful form. However, cryptanalysis uses computational methods to reveal meaning within the cipher text when a cipher key is not available. Although a thorough explanation of QC is beyond the scope of this study, a general introduction is provided for readers not familiar with the core concepts. Readers interested in a thorough explanation of QC are referred to Gisin, Ribordy, Tittel, and Zbinden’s article “Quantum Cryptography” [14]. Researchers have stated that QC “lies at the intersection of quantum mechanics and information theory” [14].

In 1948, Claude Shannon’s information theory operationalized information by establishing the bit (a binary character, e.g., 0 or 1) as the computational correlate to the lowest unit of data that can be exchanged between a sender and receiver via a channel in a communication system [15]. In contrast, quantum mechanics is a leading theory in physics that was established to explain the observed behavior of matter at the subatomic level. Researchers merged these two powerful theories, and the term ‘quantum cryptography’ was first used in 1982 [16]. Two quantum mechanical phenomena are particularly important for QC: superposition and entanglement.

In a classical system, particles can be said to be in a particular state, and particles can shift from one state to another along successive intermediate states. However, quantum superposition postulates that particles are in all possible states at once [17]. The classical deterministic notion of computing involves a system that starts from a set of input states with a corresponding label that systematically changes according to a series of discrete and sequential algorithmic steps toward an output state, and the ending output label is a measurable function of the starting input label [18]. In quantum computing, bits are replaced with quantum bits (or qubits), thereby changing the classical notion of the bit, which is in a state of 0 or 1, to the quantum bit, which can be in both states simultaneously (i.e., superposition), thereby increasing the quantum bit’s information value. Therefore, quantum computing can handle more difficult cryptographic tasks at a greater speed than the classical model [19]. Nevertheless, many challenges have yet to be overcome to realize the potential of quantum computing. For example, quantum systems are extremely delicate, and even subtle environmental effects cause decoherence (i.e., the unravelling of the quantum state). Therefore, if a fully realized physical quantum computer were to be created, its engineers would need to take extreme measures to protect the quantum system from environmental influences.

Quantum entanglement supposes that a set of particles can be described only in terms of an overall quantum system in which each particle is bound to the other regardless of the space-time barrier between them. Furthermore, interference with one particle’s state will inextricably impact the other particle’s state [17]. The entanglement phenomenon can be used for quantum teleportation, or the transmission of a message between a set of entangled particles [19]. For example, take two distinct but entangled particles (particles A and B) that are placed at a distance from one another. If a sender encodes a message into particle A by manipulating its state, then they have also instantaneously manipulated particle B’s state by virtue of quantum entanglement. A receiver at particle B can subsequently decode and obtain the message. In this case, the information was mapped onto particle A and transmitted through the quantum channel to particle B.

Wave function collapse is another concept of interest because of its cryptographic applications, and it occurs when an observer interferes with a particle in superposition. The simple act of observing a particle in quantum superposition (i.e., the particle is in all states simultaneously) collapses the particle’s superposition to a single observable state [20]. In a quantum cryptographic system, if A wants to send a secret message to B but C eavesdrops on (i.e., observes) the message before it reaches B, then C’s eavesdropping interferes with the message in such a way that C’s presence is detected.

### Literature review

Discussions among experts have indicated the growing similarity of China’s S&T research publishing practices to those of traditionally prolific countries, indicating that China’s published research is conducive to scientometric analyses [21]. Several studies have performed scientometric analyses of China’s research. Such studies typically focus on the specific development of certain S&T research topics (e.g., computer science) or adopt a higher-level view and analyze the development of S&T research in China as a whole. Previous research performed by the present author identified a dramatic increase in information security research activity emanating from China [22,23]. Moreover, researchers using scientometrics have further narrowed the scope of analysis from the broader domain of information security to cryptology, which is an information security research specialty aimed at studying the making (cryptography) and breaking (cryptanalysis) of secure codes [24]. Moving to an even lower level of analysis, other researchers have applied scientometrics to the cryptology subspecialty, cryptography [25]. Prior research that has used scientometrics to study the general topic of cryptology [26] did not focus on China, and in another case [24] restricted the data collection to the *Journal of Cryptology*, a prominent journal in the field [27]. In another article, publication output was used to study the cryptographic research specialty [25]. Neither study focused on China or QC specifically.

Other studies have argued for the need to examine China’s S&T development from a scientometric perspective [28]. The unique nature of China as a fast rising nation in terms of S&T research makes it a particularly attractive entity for analysis. Yang et al. [29] studied China’s scientific research contributions pertaining to the national problem of “haze” (low-lying debris clouds from atmospheric pollutants), whereas Xie and Willett [30] investigated computer science research from 2000 to 2009 in China. Zhai et al. [31] and He et al. [32] analyzed China’s research on entrepreneurship, biochemistry, and molecular biology, respectively, using scientometrics. Chen et al. [33] and Xu et al. [34] plotted the direction of important medical research specialties (neuroscience and gastroenterology, respectively) for China’s policymakers. Other researchers have used scientometrics to analyze library and information science in China [35–37]. From the broader research perspective, studies utilizing scientometrics have investigated the historical shift of S&T research policy in China from a Soviet model to a market-oriented model [21]. Researchers have also used scientometrics to view the historical impact of the *China Scientific and Technical Papers and Citation Database* [38]. Several articles have reported on the application of scientometrics to study national and international research collaboration among China’s scholars [39–41]. Scientometrics has also been employed to inform broader Chinese national-level research and development policy through the use of evaluative bibliometrics [9].

## Materials and methods

### Data source and collection

Compared with previous cryptology research in which data from only a single reputable Western journal were collected and analyzed [24], the current study widened the international coverage using a broader data pool that covers all major journals in the field. Data for the present study were collected from Elsevier’s *Scopus*, one of the two major sources that aggregates international research journal publication metadata. Researchers have reported that *Scopus* is stronger than the *Web of Science*, the second of the two aforementioned sources, in terms of S&T and global coverage [42]. QC research is assumed to be represented in the research data contained in *Scopus*. The data were gathered on March 15, 2017. The *Scopus* search was limited to query-only record titles, keywords, and abstracts. Similar to previous research [25,43], the current study employed several iterations of keyword combinations (including “Quantum AND Cryptograph*”) before determining that the use of “Quantum Cryptography,” a keyword identified by the *Scopus* keyword filter list, presented the optimal precision and recall. The resultant dataset included 5,923 records and was further refined to minimize noise by filtering for records from articles and proceeding papers only because books are not a common output for research on QC and reviews provide only a secondary view. In addition, the data were limited to records spanning the period from 2001 to 2017, which resulted in 4,114 records. Another dataset containing 1,477 records that was refined from the former dataset included only records pertaining to China’s research, not including Hong Kong and Taiwan due to their differing political and economic systems. The data were exported to the.RIS file format and included all bibliographic metadata fields. The data underwent cleansing and disambiguation via the automated character string similarity distance detection functionality of the science mapping application *SciMAT* [44] combined with several iterations of a manual review.

### Data analysis

The analysis was performed over the course of several days shortly after data collection. All calculations utilized integer counting, which researchers have found to be appropriate for country-level analyses, instead of fractional counting [45]. For RQ 1, the unit of analysis was the contributing countries. For RQ 2, the larger dataset that contained all countries was analyzed using several performance measures, whereas the China-specific dataset was scrutinized using *SciMAT* to develop co-word cluster network visualizations depicting the evolution of research themes. *SciMAT* is an open source software application designed to support the full range of procedures involved in bibliometric science mapping, including data cleansing, normalization, and the production of visualizations [44]. The following paragraphs outline both sets of analysis procedures executed in the current study.

The first phase of analysis is based on recent similar research [22]. Three quantitative performance measures were used in the analysis, namely the H-index, Publication Frequency, and citation score. The primary performance measure used in this study was the H-index. The H-index has a number of advantages over other measures because it is resistant to irregular changes in citation and publication patterns and represents a single measure that accounts for both the quantitative (i.e., publication frequency) and qualitative (i.e., citation score) dimensions of impact. Although the H-index was initially created to ascertain the impact of a scientist, researchers have established its validity and reliability when used to measure the research impact of other larger units of analysis (e.g., countries) [46]. The H-index is calculated based on the following equation: “A scientist has index h if h of his/her Np papers have at least h citations each and the other (Np-h) papers have no more than h citations each [47].” Similar to previous research [48], the current study also calculated several other standard performance indicators, including publication frequency (PF) [49] and citation score (CS) (i.e., the number of times a work has been referenced in other works) [10]. These indicators provide a well-rounded country-wise outlook on the performance development of China’s QC research.

The second phase of analysis was adapted from established practice [43,44,50] and addresses the thematic evolution of China’s QC research using science mapping. In the context of this study, themes represent the specific research focus of China’s scientists in the area of QC. Themes are operationally defined and labeled based on keyword clusters derived from co-word analyses. Thematic evolution is established by plotting clusters according to segmented time periods. The use of co-words for mapping creates clusters of interconnected keywords derived from document metadata. The selected unit of analysis for the weighted undirected co-word network was words (author keywords, source keywords, and added keywords). Words carry direct semantic meaning and are more conducive to identifying research themes than are authors or citations [51]. The data were segmented into the following 4-year periods to highlight the thematic shifts over time: 1) 2001–2004, 2) 2005–2008, 3) 2009–2012, and 4) 2013–2017. The fourth segment included a fifth year that was composed of 34 documents from 2017 because data were collected in March of 2017 and the author decided that including the latest research records was important. The cluster network processing parameters were as follows: 1) a document frequency threshold of 1 for the first segment and 2, 3, and 4 for the remaining three segments was set to limit word inclusion; 2) co-occurrence (i.e., in this case, the frequency by which words appear together in a document) was selected as the matrix preference; 3) the inclusion value threshold of the first segment’s network edges was set to 1 and the latter three segments were set to 2, 3, and 4; 4) the equivalence index was chosen as the normalization measure; 5) the simple centers clustering algorithm [50] was used with a maximum network size of 12 and a minimum network size of 3; 6) both the core and the secondary mappers were selected; 7) the H-index and the sum citation evaluative measures were selected for node display; and 8) Jaccard’s index [52] and the inclusion index [53] were chosen as measures for longitudinal and overlap mapping. The analysis produced strategic diagrams and cluster keyword network graphs of co-word research groups plotted according to time periods, centrality, and density measures. Centrality measures the frequency at which words within a specific network cluster co-occur with words from other clusters (between-cluster connections), and density measures the degree to which words within a cluster co-occur with one another (within-cluster connections) [50,54]. The cluster keyword network graphs provided the most granular view and were used to enhance the analysis but were too numerous to address in full (see S1 File).

## Results

### RQ 1

Only data related to the top 5 contributing countries (selected based on H-index) between 2001 and 2017 were included in the analysis because limiting the number of countries to 5 improves visibility and allows for a more concise analysis. The following analysis compares China to its top 5 peer countries in terms of research performance measures (see S1 Table for summarizing totals.). The following figures utilize country abbreviations derived from the IOS “ALPHA-2 Codes” (People’s Republic of China, CN; United States, US; Canada, CA; United Kingdom, UK; and Germany, DE). ISO country codes can be obtained from the official ISO website: https://www.iso.org/obp/ui/#search. In addition, the statistical median of the 5 countries for each measure was calculated and included in each figure as a benchmark.

#### Publication frequency

**Fig 1:**
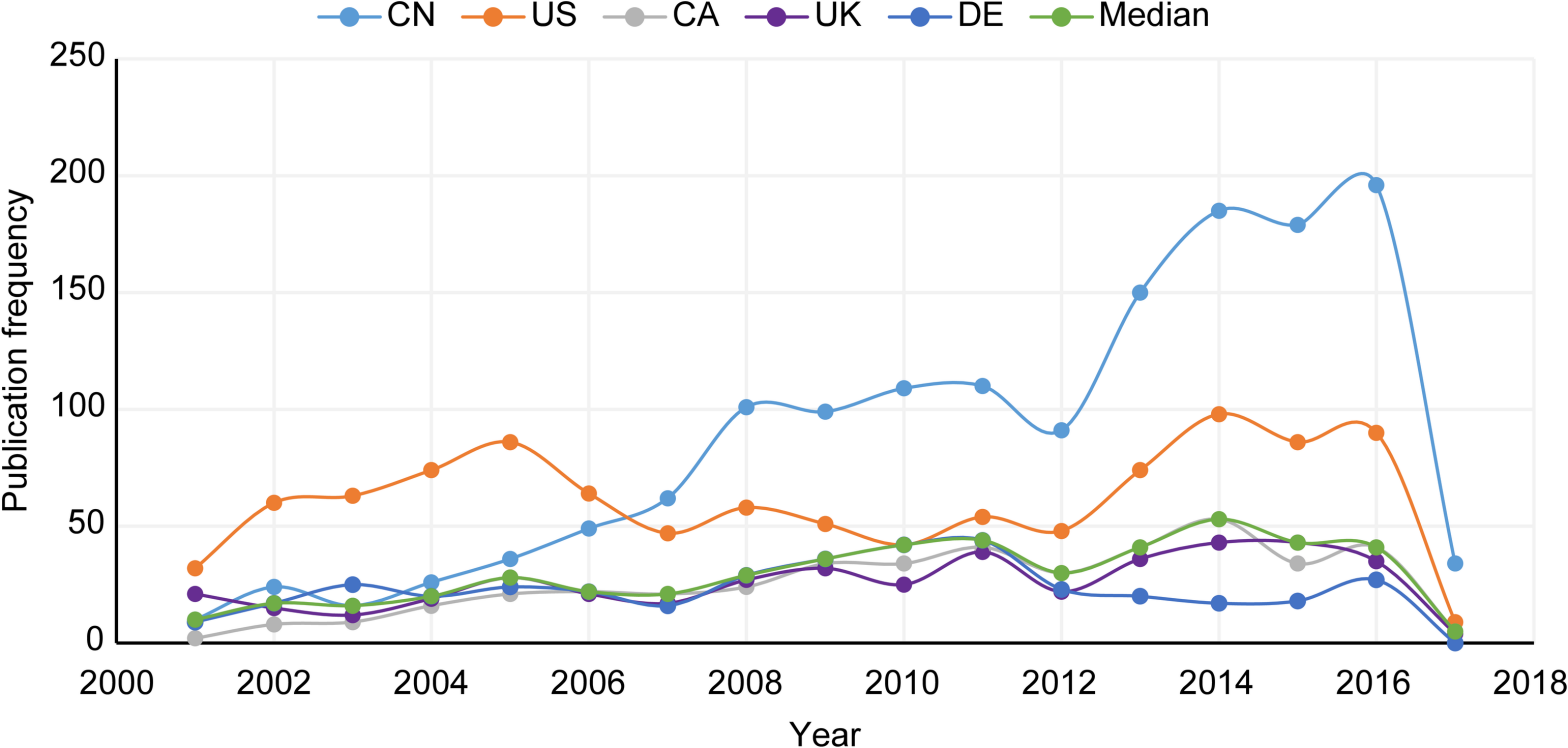
Publication frequency trends from 2001 to 2017.

Fig 1 plots the output patterns spanning from 2001 to 2017 for the top 5 QC countries based on each country’s PF (see S2 Table for PF totals). From 2001 to 2006, China was publishing at a rate lower than that of the US. Starting in 2003, however, China’s output steadily increased at an average rate of 11.5 publications per year before overtaking the US in 2007. Since then, China has been the top producer of QC research. The period between 2008 and 2012 was marked by fairly consistent output and slight oscillations. More interestingly, China experienced exponential growth (115% increase) from 2012 to 2016, which represents a 27% greater increase than the second most active country (i.e., the US) and is 78% higher than the median. At its high point (i.e., 2016), China’s output was 118% greater than that of the US. The comparison with the US is important because until China’s rise, the US had consistently been publishing at a greater rate than all other countries. China has shown dramatic changes over the 17-year study period, and at this point, China is producing QC research at a rate well beyond its peers’.

#### Citation score

**Fig 2:**
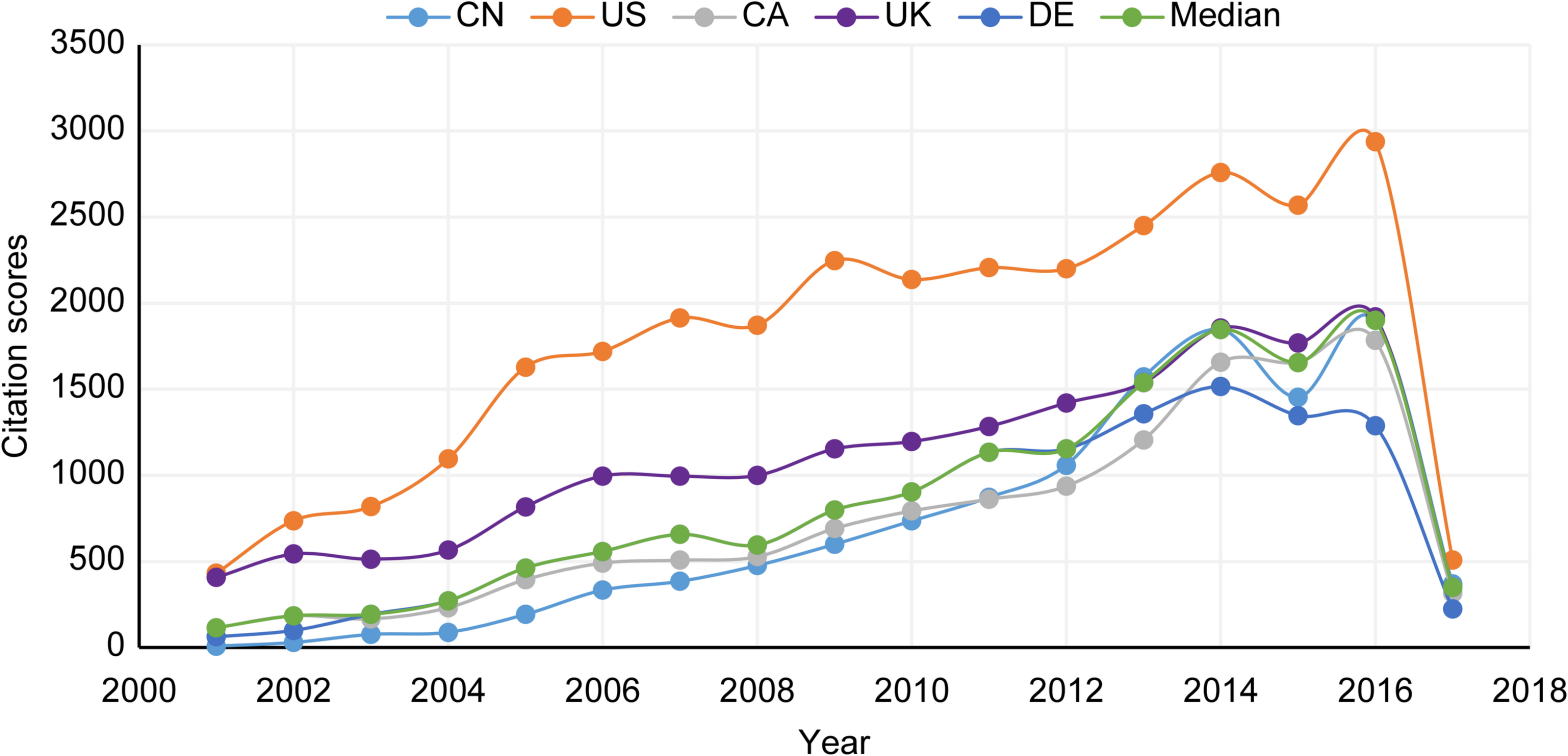
Citation score trends from 2001 to 2017.

As shown in Fig 2, the CS evolution for China is very different from the PF evolution (see S3 Table for CS totals). China’s CS was consistently below its top 5 peer countries from 2001 until 2011, when it slightly overtook CA. The US had a significant CS lead over all other countries during this 11-year period. However, during the first 11 years, China’s CS progress exceeded the median CS rate of increase by 38%. Except for an unexplained dip in 2015, China’s CS trend is set to rise beyond all but the US’s, which indicates an increase in the utilization of China’s research by QC scholars (a sign of increasing relevance).

One reason suggested by researchers for the disproportionate number of US citations is the Mathew effect, which states that researchers are inclined to cite studies produced by the US because the country’s publications are often highly cited, thereby causing compounded citation growth [55]. Although China’s PF and CS have both shown growth trends, a sizable gap between China’s PF (see Fig 1) and its CS (see Fig 2) is observed relative to that of the other top 5 countries. In other words, China is contributing proportionally more QC research than its peers, but that research is not being acknowledged via citations at the same rate. Nevertheless, this pattern is clearly changing, as shown by the difference between China’s CS between 2006–2011 (below its peer top 5 countries) and 2012–2016 (climbing above several of the other top 5 countries) as shown in Fig 2. In addition, the CS is not a static value, and future researchers may find previous Chinese QC publications valuable for their work, thereby increasing the CS of past Chinese QC research publications. Thus, as China continues to show marked success with its QC research, it may also experience compounded citation growth as is the case with the US and suggested by the Matthew effect.

#### H-index

**Fig 3:**
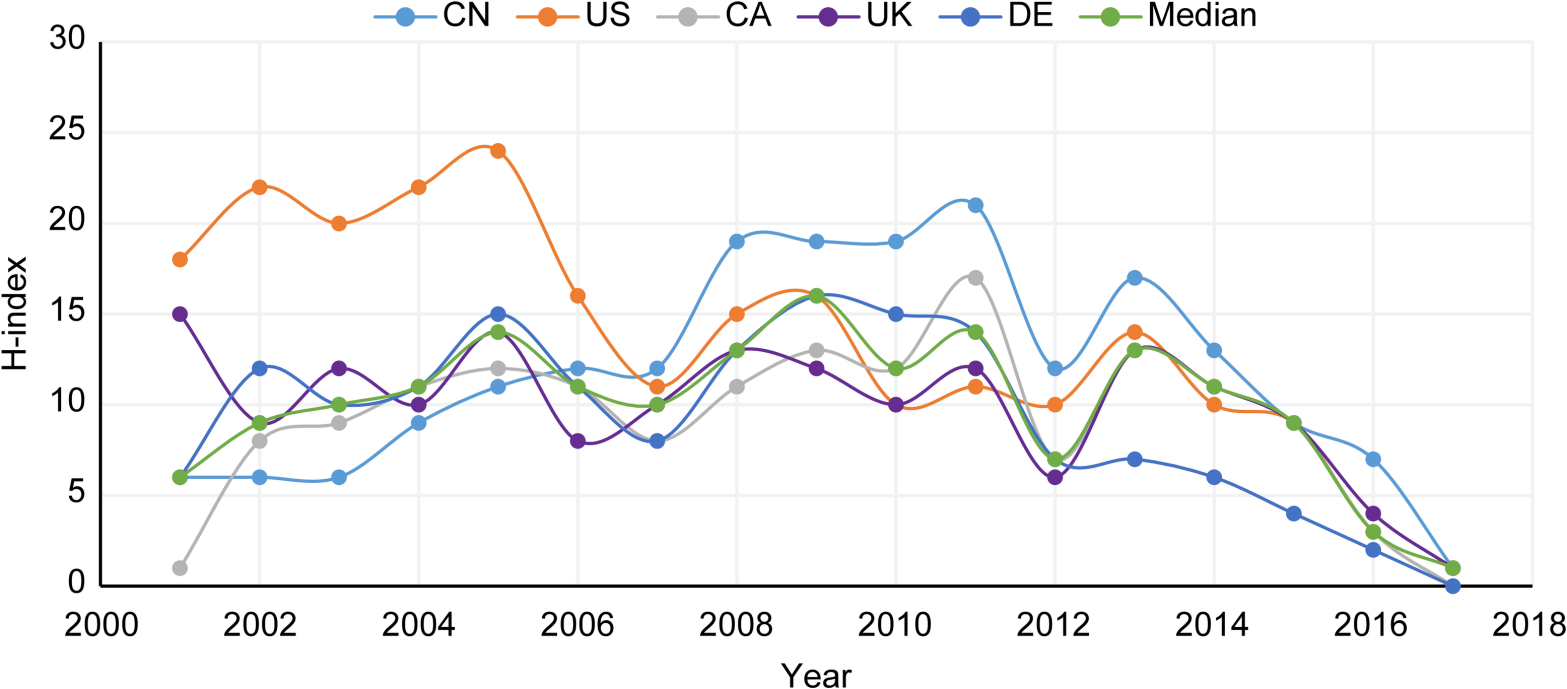
H-index trends from 2001 to 2017.

According to Fig 3, which utilizes the H-index (a more robust measure of impact than CS), China was below all but CA between 2001 and 2005 (see S4 Table for H-index totals). Furthermore, after 2006, China had the greatest H-index, and then from 2007–2008, China displayed a sharp rise (58% increase) before maintaining its role as a leading country for the remainder of the period. The H-index can overcome many of the limitations posed by previous measures (i.e., PF and CS), and it clearly illustrates a shifting of the lead country status from the US (in the former portion of the period) to China (in the latter portion of the period).

#### Collaboration frequency

**Fig 4:**
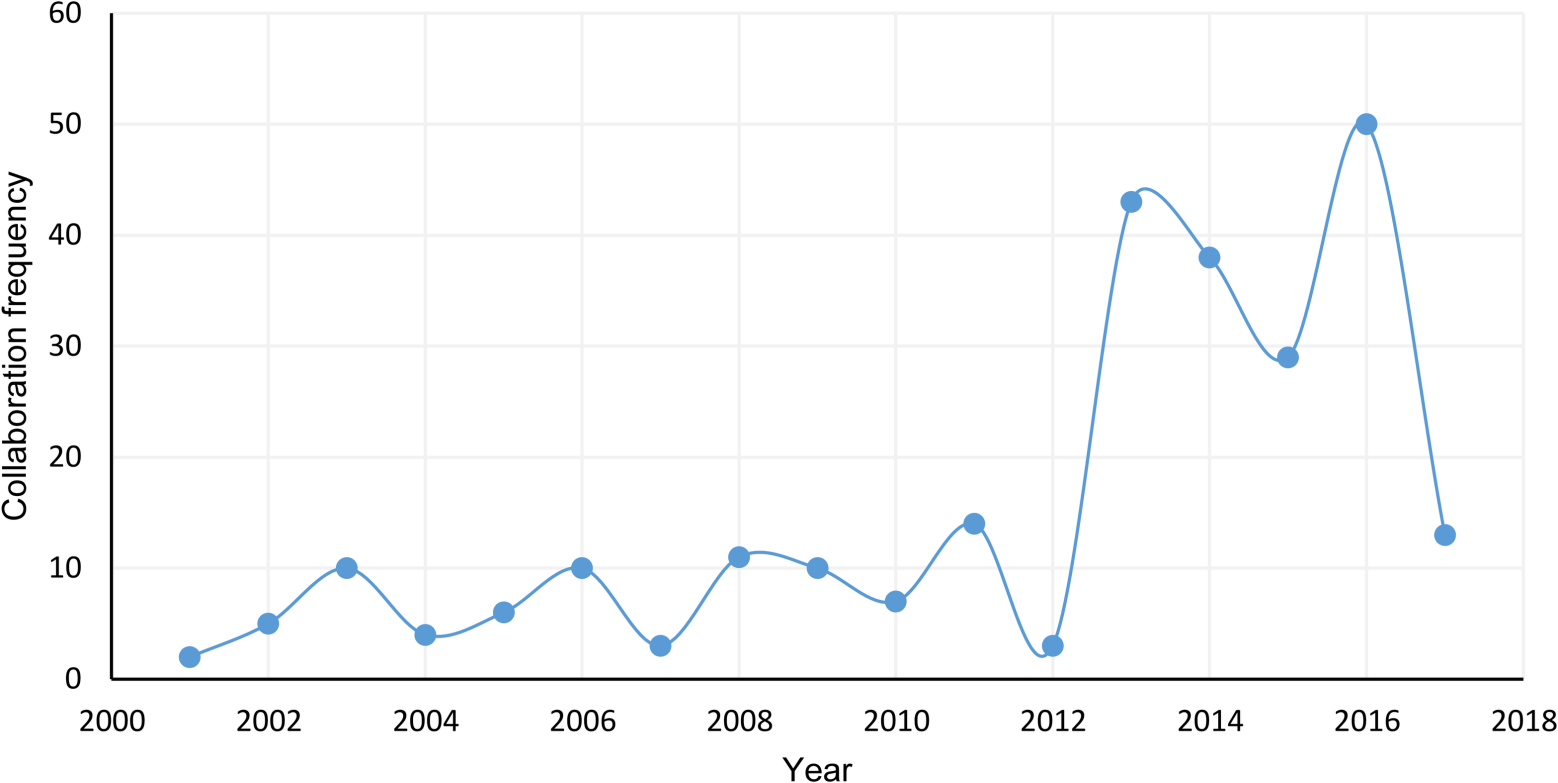
Collaboration frequency from 2001 to 2017.

Fig 4 shows China’s global research collaboration efforts between 2001 and 2012 as oscillating in a steady low-positioned pattern, not exceeding 14(see Fig 4 and S5 Table). Interestingly, collaboration between 2012 and 2013 increased by a factor of 14 and then dropped 48% between 2012 and 2015. The period ended with a dramatic 72% increase between 2015 and 2016.

#### Total collaboration partner frequency from 2001 to 2017

Table 1 shows the total frequency at which countries collaborated with China (limited to the top 11 for brevity) on QC research publications between 2001 and 2017.China has collaborated with the US on QC research more than any other country.

**Table 1:** Top 11 collaborating countries.

Countries/Territories	Collaboration Partner Frequency
United States	46
Australia	32
Canada	30
Hong Kong	19
United Kingdom	15
South Korea	14
Singapore	13
Japan	12
Germany	11
Portugal	8
France	8

### RQ 2

#### Thematic evolution as shown in strategic diagrams

Strategic diagrams plot themes (circles) according to centrality and density. Each circle is proportionately sized and numbered according to the frequency of documents in which its keywords can be found, and the results are referred to as the document frequency (DF). As described in prior research [50], strategic diagrams can be interpreted based on four quadrants: 1) the top-left quadrant shows “highly developed and isolated themes … well-developed internal ties, but unimportant external ties … [that are] marginally important to the field … specialized and peripheral in character,” 2) the bottom-left quadrant indicates “emerging or declining themes … [that are] weakly developed and marginal;” 3) the top-right quadrant notes motor themes that are “well developed and important for structuring of a research field … [and] related externally to concepts applicable to other themes that are conceptually closely related;” and 4) the bottom-right quadrant highlights “basic and transversal themes [keywords used only in a single period] … [that are] important for a research field, but are not developed.” In addition to the strategic diagrams, tables for each period are also presented, which contain quantitative and qualitative performance measures to assist with the theme analysis.

**Fig 5:**
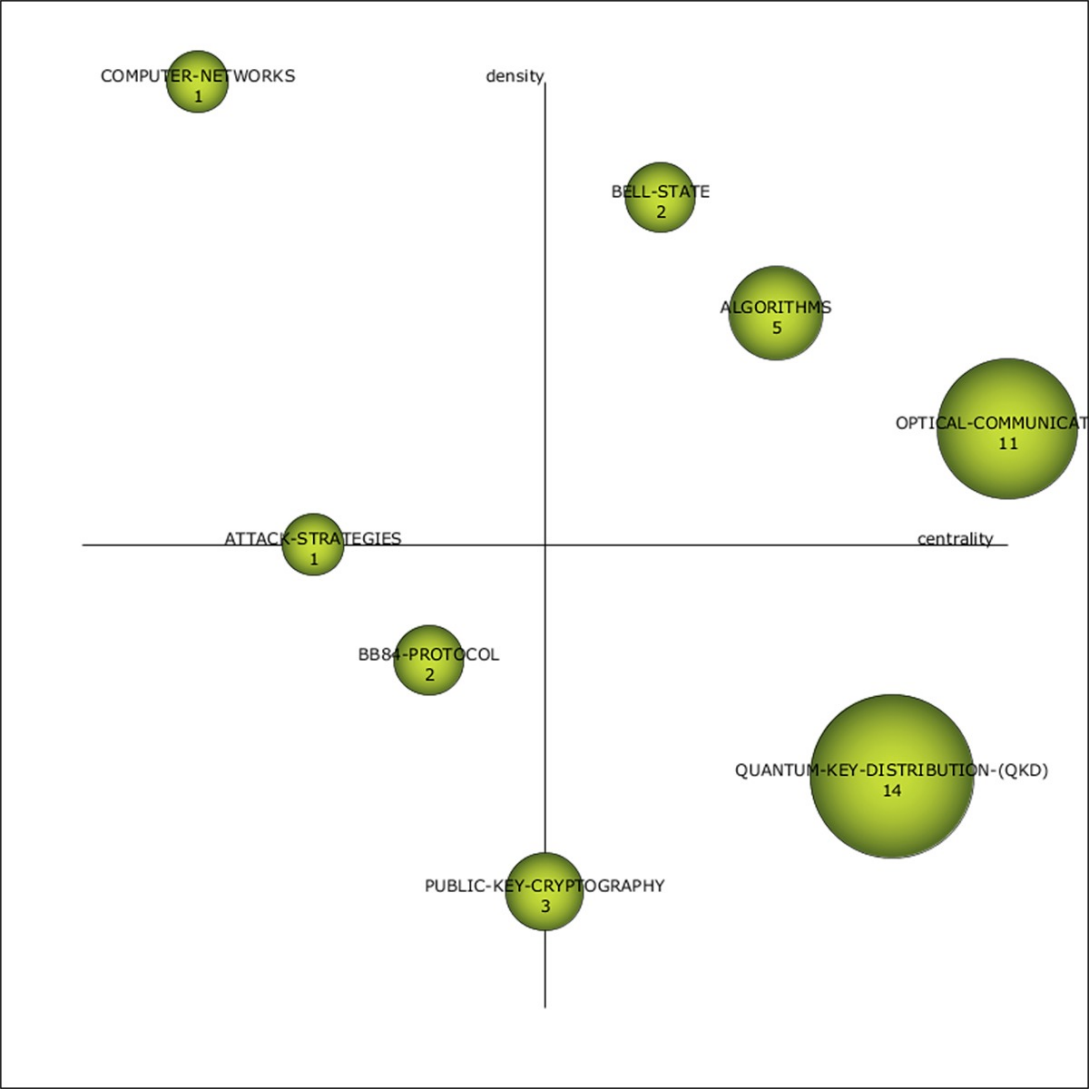
Quantum cryptography strategic diagram from 2001–2004.

The 2001–2004 period is marked by a low document count (76) and few overall keywords (86), meaning that at this stage, China’s scientists were not focused on QC research. According to Fig 5 and Table 2, the early period from 2001–2004 shows two main themes: QUANTUM-KEY-DISTRIBUTION-(QKD) and OPTICAL-COMMUNICATION. The QUANTUM-KEY-DISTRIBUTION-(QKD) theme has a notably high CS (1,497), which illustrates its relevance and importance to the specialty (see Table 2), whereas the OPTICAL-COMMUNICATION theme’s spatial position (based on centrality and density measures) in the top-right quadrant suggests that it is structurally important and well connected to other themes.

**Table 2:** Theme performance measures for the period 2001–2004.

Name	DF	H-index	CS	Centrality	Density
QUANTUM-KEY-DISTRIBUTION-(QKD)	14	6	1,497	75.53	38.94
OPTICAL-COMMUNICATION	11	1	8	86.17	71.5
ALGORITHMS	5	3	51	27.28	71.99
PUBLIC-KEY-CRYPTOGRAPHY	3	1	52	11.68	0.12
BELL-STATE	2	1	28	24.35	0.88
BB84-PROTOCOL	2	1	4	10.92	50
COMPUTER-NETWORKS	1	1	102	4.91	100
ATTACK-STRATEGIES	1	1	1	10.12	0.5

**Fig 6:**
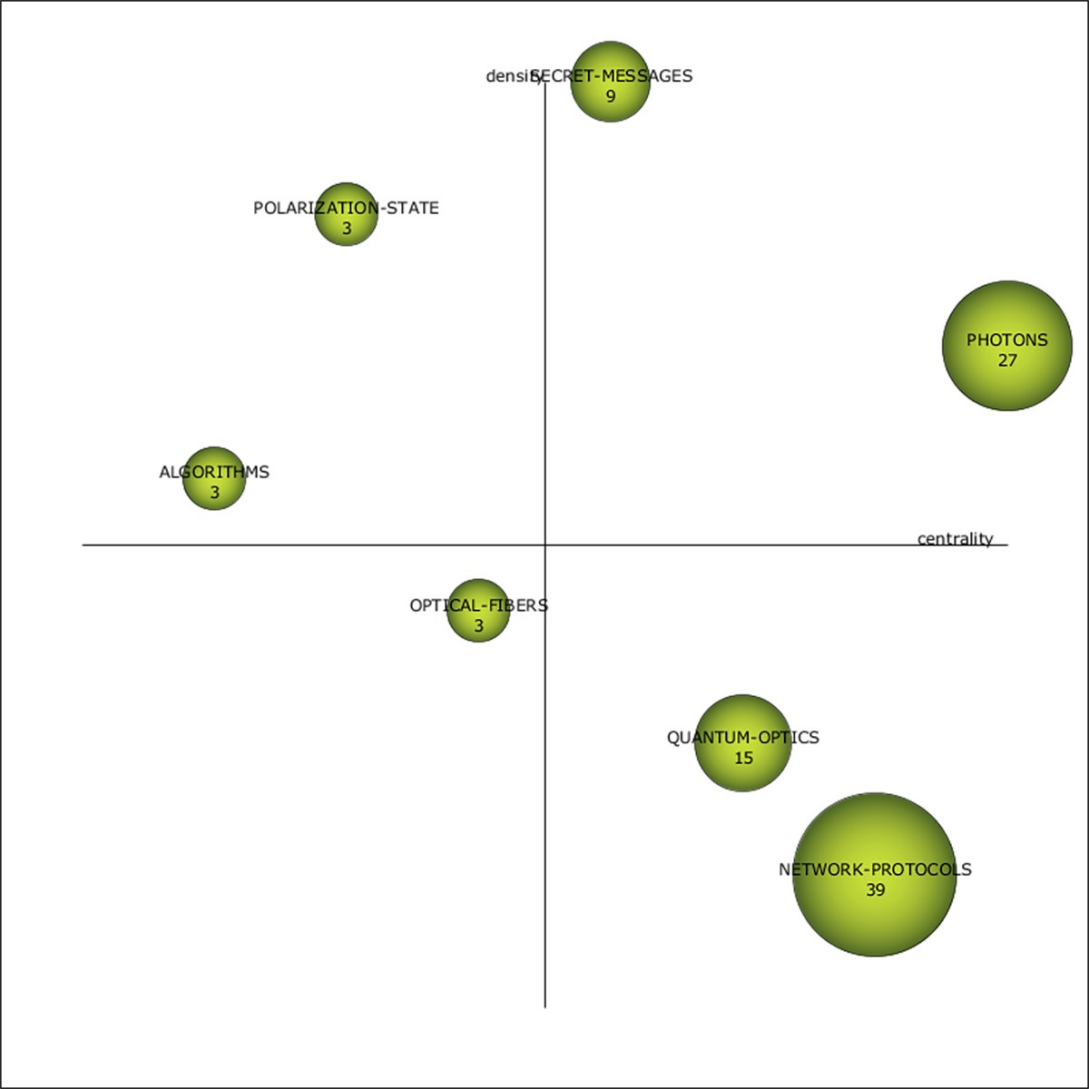
Quantum cryptography strategic diagram from 2005–2008.

During the 2005–2008 period, China dramatically increased its QC research activity, the number of documents increased from the previous period to 248 (a 226% increase), and the overall number of keywords grew to 239 (a 178% increase), although the number of themes decreased from the previous period (see Fig 6). The most active theme between 2005 and 2008 (Fig 6) is NETWORK-PROTOCOLS. NETWORK-PROTOCOLS is positioned as a transversal theme. The PHOTONS theme appears to be well developed, and the OPTICAL-FIBERS theme appears to be an emerging focus. The themes PHOTONS, QUANTUM-OPTICS, and NETWORK-PROTOCOLS thus had the greatest impact on the specialty during the 2005–2008 period (see Table 3).

**Table 3:** Theme performance measures for the period 2005–2008.

Name	DF	H-index	CS	Centrality	Density
NETWORK-PROTOCOLS	39	8	190	21.12	0.14
PHOTONS	27	8	375	36.2	29.35
QUANTUM-OPTICS	15	5	365	14.9	8.29
SECRET-MESSAGES	9	2	51	9.02	76.16
OPTICAL-FIBERS	3	3	133	7.51	8.33
ALGORITHMS	3	3	64	0	16.67
POLARIZATION-STATE	3	2	8	6.08	33.33

**Fig 7:**
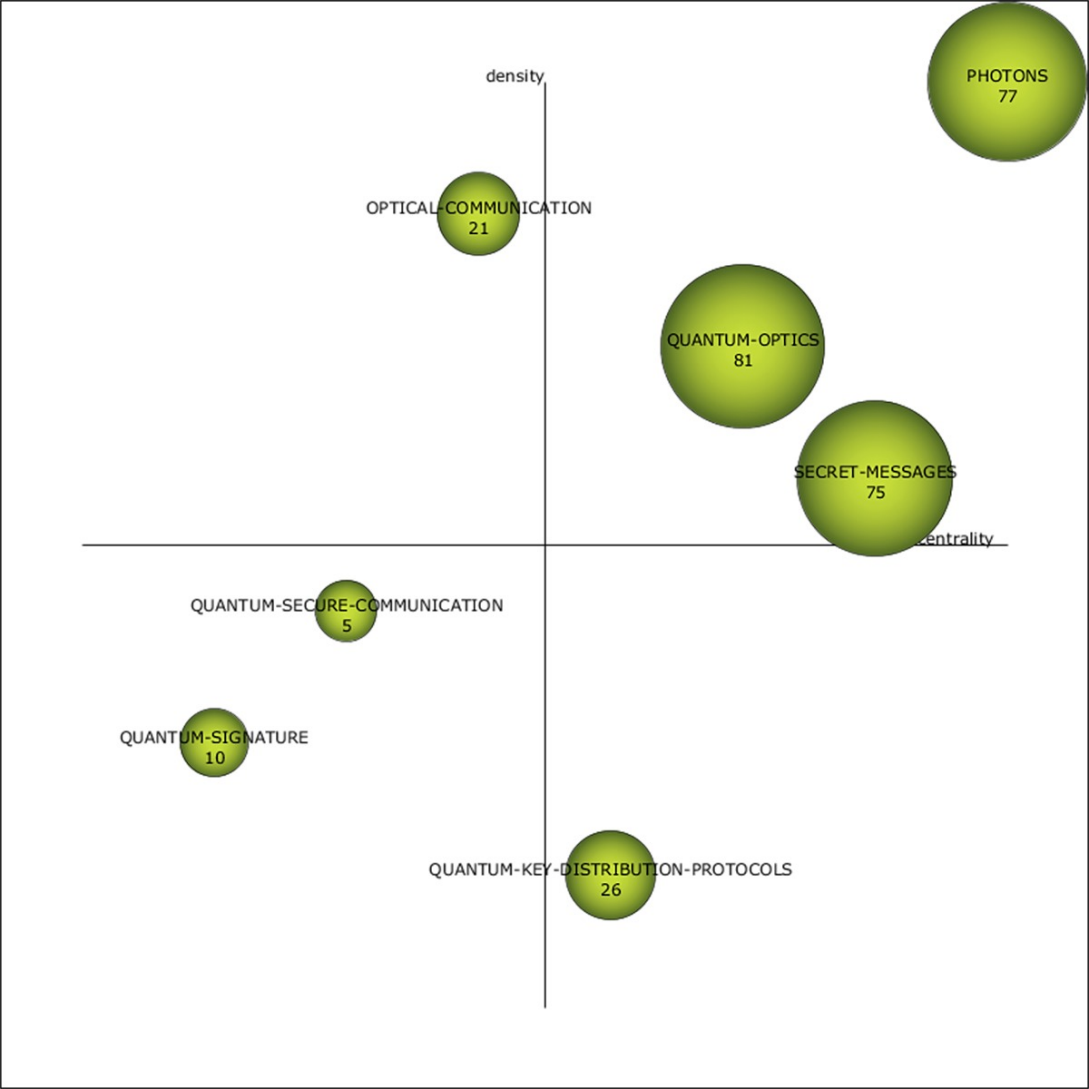
Quantum cryptography strategic diagram from 2009–2012.

A 65% increase in documents and a 34% increase in overall keywords occurred from the 2005–2008 period to the 2009–2012 period, which was lower than the previous increase. As indicated in Fig 7, three important motor themes (top-right quadrant) are all measured as high performing (see Table 4). The PHOTONS theme has been carried over from the previous period but maintains the same position. The motor theme labeled SECRET-MESSAGES, which was also a carryover and is located in the same quadrant as before, displays the greatest impact measures but has increased in centrality from the previous period. Although the QUANTUM-OPTICS theme was carried over from the previous period, it has shifted quadrants from the bottom left in the 2005–2008 period (indicating it was a basic and transversal theme) to the top right in the 2009–2012 period (indicating it was a foundational theme with ties to other specialty themes) and presents significantly greater performance measures. Although the QUANTUM-KEY-DISTRIBUTION-PROTOCOLS theme, which is located in the bottom-right quadrant, does not have a significant impact, it has been resurrected in part (i.e., QUANTUM-KEY-DISTRIBUTION) from an earlier 2001–2004 period theme (i.e., QUANTUM-KEY-DISTRIBUTION-(QKD)). Positioned in the bottom-left quadrant of Fig 7 are the emergent themes QUANTUM-SIGNATURE and QUANTUM-SECURE-COMMUNICATION. The OPTICAL-COMMUNICATION theme (top left) appears isolated and developed.

**Table 4:** Theme performance measures for the period 2009–2012.

Name	DF	H-index	CS	Centrality	Density
QUANTUM-OPTICS	81	19	952	38.34	16.67
PHOTONS	77	15	720	61.22	48.3
SECRET-MESSAGES	75	21	1,166	43.81	15.75
QUANTUM-KEY-DISTRIBUTION-PROTOCOLS	26	6	198	18.46	3.07
OPTICAL-COMMUNICATION	21	7	214	12.06	31.62
QUANTUM-SIGNATURE	10	4	110	1.71	7.65
QUANTUM-SECURE-COMMUNICATION	5	4	132	3.86	11.4

**Fig 8:**
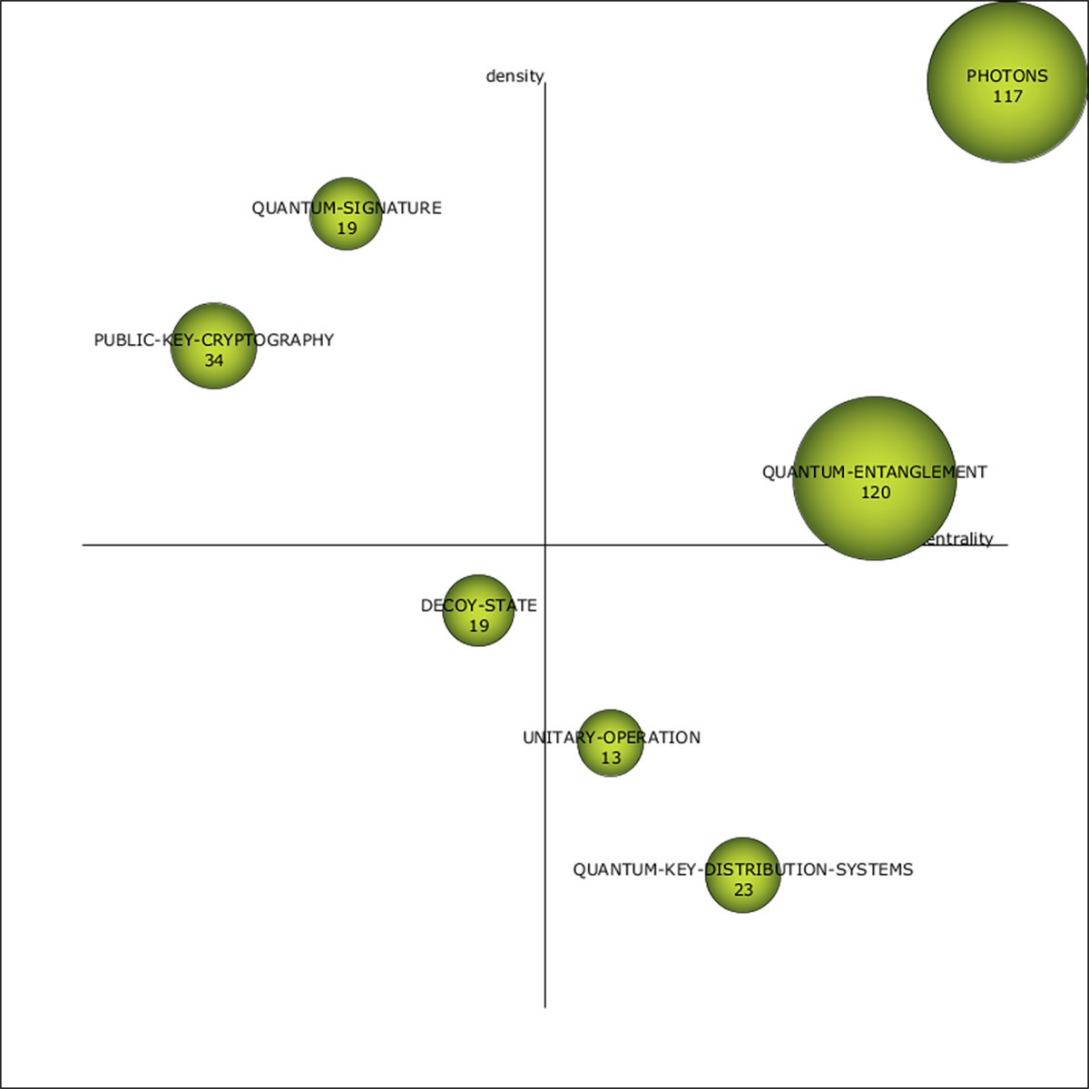
Quantum cryptography strategic diagram from 2013–2017.

All but the first period maintained seven themes. The period 2013–2017 exhibited an 82% increase in documents (744) and a 29% increase in overall keywords (413) from the previous period. As illustrated in Fig 8, the theme PHOTONS (top-right quadrant) appears again as a high-impact and dense theme and as the most central motor theme. The other motor theme, QUANTUM-ENTANGLEMENT, has only recently emerged and leads with respect to quantity and impact measures (see Table 5). The top-left quadrant of Fig 8 reveals two highly developed and isolated themes: QUANTUM-SIGNATURE (a carryover theme from the previous period) and PUBLIC-KEY-CRYPTOGRAPHY (a theme resurrected from the 2001–2004 period). The bottom-right quadrant displays QUANTUM-KEY-DISTRIBUTION-SYSTEMS as a basic and transversal theme.

**Table 5:** Theme performance measures for the period 2013–2017.

Name	DF	H-index	CS	Centrality	Density
QUANTUM-ENTANGLEMENT	120	10	394	24.27	8.74
PHOTONS	117	8	253	24.66	22.88
PUBLIC-KEY-CRYPTOGRAPHY	34	3	41	0.69	17.17
QUANTUM-KEY-DISTRIBUTION-SYSTEMS	23	3	38	4.39	3.47
QUANTUM-SIGNATURE	19	5	91	1.72	22.5
DECOY-STATE	19	5	42	2.5	8.06
UNITARY-OPERATION	13	5	69	3.1	4.11

#### Keyword evolution from a quantitative perspective

**Fig 9:**
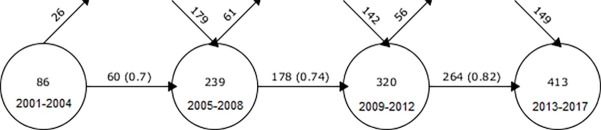
Overlapping map showing a quantitative view of keyword evolution.

Fig 9 illustrates the quantitative transition and exchange of keywords from one period to the next. Starting from the earliest period on the left side (2001–2004) and moving to the latest period on the right side (2013–2017), each circle in Fig 9 identifies a particular period along with the number of keywords it contains. Periods 1, 2, 3 and 4 had total keyword counts of 86, 239, 320, and 413, respectively. Moreover, the arrows moving from one period to the next show the number of keywords exchanged (integer value) and the fraction of overlap (i.e., Similarity Index) in parentheses [50]. The arrows moving into the circles from above express keywords that are new to the period (not carried over from the previous period), whereas the arrows exiting from the tops of the circles indicate the number of keywords that are not used in the subsequent period. According to Fig 9, the number of keywords steadily increased from period to period. Moreover, the number of keywords for the last period was four times greater than that for the first period. Significant keyword overlap is observed, which indicates the continuity of core themes.

#### Thematic evolution from a co-word cluster network perspective

**Fig 10:**
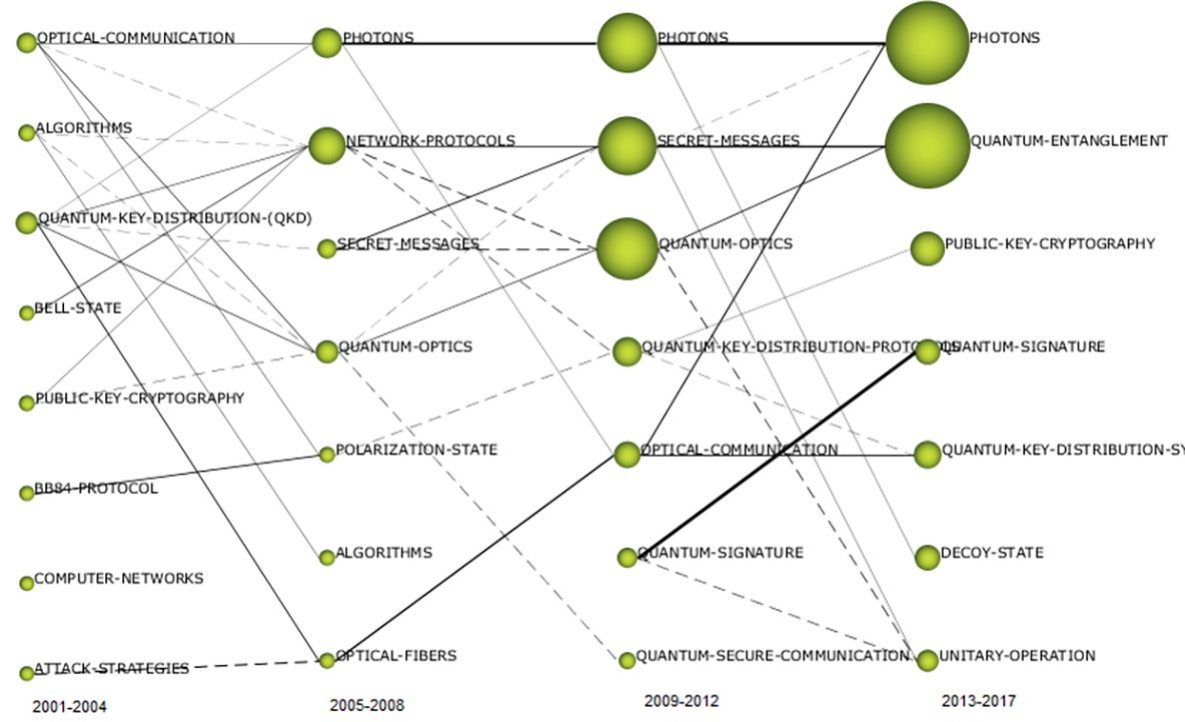
Evolution of quantum cryptography themes from 2001–2017.

The next graph (Fig 10) plots each period in columns (earliest period on the left side and latest period on the right side). This layout allows the viewer to track shifting keyword patterns over time. Nodes represent themes, and theme clusters are composed of several networked keywords (see S1 File). The node size is proportional to the frequency at which keywords within the theme occur in documents (greater frequencies correspond to larger circles). Themes that have a relationship between periods are connected by links. Link thickness is proportional to the extent to which the two nodes have a commonly shared element. Two different link types are observed: 1) solid links indicate a direct theme label carryover (each node is labeled with the same label or shares part of the label) and 2) broken links illustrate that the two themes share keywords in their respective clusters that are different from the keywords in their labels [50].

The following analysis investigates the thematic evolution of China’s QC research by studying the themes in each of the time periods, including each theme’s keyword composition, while also reviewing their thematic transition across the total continuous timeline as shown in Fig 10. Two themes stand out in the transition from the first time period to the second in terms of numerous strong ties: QUANTUM-KEY-DISTRIBUTION-(QKD) and OPTICAL-COMMUNICATION. The ALGORITHMS theme also has several ties, although most are weak. Of the former two themes, China’s scientists primarily focused on QUANTUM-KEY-DISTRIBUTION-(QKD) (DF: 14), and this theme also had the greatest impact (CS: 1497). Although the QUANTUM-KEY-DISTRIBUTION-(QKD) theme did not have any direct carryover between the first two periods, its strong ties to the themes of the 2005–2008 period (PHOTONS, NETWORK-PROTOCOLS, QUANTUM-OPTICS, and OPTICAL-FIBERS) suggest that it was a foundational theme for much of the latter period’s research. Comparatively, the impact of OPTICAL-COMMUNICATION was significantly reduced (CS: 8), although it had strong connections to PHOTONS, QUANTUM-OPTICS, and POLARIZATION-STATE. In the 2005–2008 period, NETWORK-PROTOCOLS and QUANTUM-OPTICS acted as thematic bridges between periods because of their high degree of connectedness (see Fig 10).

#### Themes appearing in several continuous periods

Fig 10 illustrates how the most reoccurring theme, PHOTONS, spanned from the 2001–2004 period to the 2013–2017 period. In all three of the periods in which the PHOTONS theme was present, it maintained the second highest performance measures, indicating its significant role in China’s QC research. Although the ALGORITHMS theme carried over from the 2001–2004 period to the 2005–2008 period, it received little effort and had minimal impact (see Tables 2 and 3). Both the SECRET-MESSAGES and QUANTUM-OPTICS themes carried over from the 2005–2008 period to the 2009–2012 period, and each showed significant increases in terms of effort and impact measures (see Tables 3 and 4). The QUANTUM-SIGNATURE theme was present in both the 2009–2012 period and the 2013–2017 period, although the performance measures were relatively low.

#### Disappearing themes

The COMPUTER-NETWORKS theme was observed only early in the 2001–2004 period and had no ties. Other early disappearing themes (i.e., ATTACK-STRATEGIES and BELL-STATE) also had few ties and low performance measures (see Table 2 and Fig 10). POLARIZATION-STATE was a disappearing theme from 2005–2008 and also had low performance measures but several ties (see Table 3 and Fig 10). The PUBLIC-KEY-CRYPTOGRAPHY theme disappeared after the 2001–2004 period only to be resurrected in the 2013–2017 period. Similarly, QUANTUM-KEY, which was part of several themes, was observed as a prefix for themes in different periods.

#### Recently emerging themes

Of the newly emerging themes, QUANTUM-ENTANGLEMENT has received the greatest amount of effort and has the greatest impact (see Table 5). The other emergent themes (i.e., DECOY-STATE and UNITARY-OPERATION) are relatively low in terms of performance (see Table 5), although the UNITARY-OPERATION theme is linked to several important themes from the 2009–2013 period (i.e., QUANTUM-OPTICS, SECRET-MESSAGES, and QUANTUM-SIGNATURE).

## Discussion

### RQ 1

The first research question asks how China’s international QC research performance is evolving compared with that of other countries. China has grown to outperform its international peers in QC research. Relative to the other top 5 countries, China ranked first in PF and the H-index over the past decade. China’s increasing QC research capabilities are in line with China’s increased emphasis on funding and developing infrastructure to support quantum information science [1]. With regard to the CS, China’s progress was well beyond the annual median increase. Moreover, China’s CS increased to a level consistent with that of the other top countries (not including the US) in 2011 and rose above 3 of the 5 top countries in 2013. After 12 years (2001–2012) of low collaboration, China showed significantly increased research collaboration over the remaining 4 years (2013–2016), which indicates that China’s QC research performance evolution involves increased output as well as increased collaboration and quality. Researchers have found that research collaboration can positively impact other performance measures [56], and the Chinese government has made it a point to encourage collaboration [57], potentially driving performance. These findings clearly suggest that China has taken the role of lead nation.

### RQ 2

The second research question asks how China’s main QC themes have evolved. Considering China is the premier driving force behind international QC research, an understanding of China’s QC research themes can provide clarity into how QC technologies will take shape and how they will impact the cyber domain. To address the second research question, the quantitatively derived co-word maps were analyzed in conjunction with a qualitative subject matter review of pertinent core literature to develop context. This process identified China’s main thematic progression through the various time periods. Several main themes stood out because of their high performance measures, degree of connectedness, and meaning in the context of the QC specialty. Thus, 4 main themes were developed from the review: QUANTUM-KEY-DISTRIBUTION, PHOTON-OPTICAL-COMMUNICATION, NETWORK-PROTOCOLS and QUANTUM-ENTANGLEMENT. The following explanation of each theme coincides with the chronological development of China’s QC specialty. However, significant overlap occurred throughout the entire timeline.

First, QUANTUM-KEY-DISTRIBUTION, which is a central quantum cryptographic task, exploits quantum mechanical properties to create a secure information-theoretic solution to the problem of key exchange [58], which involves privacy and confidentiality challenges that occur when a cipher key is securely transferred between two parties and an eavesdropper executes a man-in-the-middle attack. QUANTUM-KEY-DISTRIBUTION allows two parties to communicate in such a way that if an eavesdropper listens in, regardless of how quietly, the mere presence of the third party will be detectable. The QUANTUM-KEY-DISTRIBUTION theme was a common topic for international QC researchers during the early period, and it encapsulates several other themes (e.g., PUBLIC-KEY-CRYPTOGRAPHY, SECRET-MESSAGES, etc.).

Second, PHOTON-OPTICAL-COMMUNICATION involves QC’s use of photonic methods and techniques during the experimental development of QC systems [14]. Moreover, QC researchers have attempted to overcome certain challenges experienced when photons are exchanged across a medium (e.g., optical fiber) with, for example, the creation of a photon gun. Science maps appear to show that China has expended significant effort on the PHOTON-OPTICAL-COMMUNICATION theme, which indicates that applied research is a consistent goal.

From a higher-level perspective, the third theme, NETWORK-PROTOCOLS, broadly covers research on the many protocols that underpin QC research (e.g., BB84 protocol, 2-State Protocol, EPR Protocol, etc.) [14]. These protocols involve the formal rules developed by QC researchers that dictate how the QC system operates.

Lastly, QUANTUM-ENTANGLEMENT, which is the quantum mechanical state in which two particles that are bound can influence each other across space-time barriers, can be interpreted in two ways. First, researchers study weaknesses in QC protocols using a quantum variation of the traditional man-in-the-middle attack (i.e., the Einstein–Podolsky–Rosen attack) by exploiting the quantum entanglement paradox [14,59]. Second, researchers have also used the affordances provided by quantum entanglement to develop secure quantum teleportation protocols for a quantum version of the one-time-pad [14]. In other words, two parties can share a message in the form of a quantum state, and even if a third party has the ability to eavesdrop, nothing can be learned of the message.

### Limitations of the study

The use of scientometrics presents several drawbacks and methodological limitations noted by previous researchers [60] as primarily related to the use of bibliometric data [19]. First, the coverage of research publications from developing countries and languages other than English may be under represented in *Scopus*. Second, peer-reviewed scientific research articles have a lag time of approximately nine to 18 months before an article is published. However, conference papers are published considerably faster, and technical research domains, such as QC, tend to publish conference papers. Third, citation data can be skewed by citation bias, negative citations, and self-citations. However, researchers [61,62] have reported that a large sample size reduces the impact of such disparities. The length of time it takes for new publications to be citated also presents an unmitigated limit of this study. Fourth, the scope of this study specifically aimed at collecting data on QC research, and such data may also exist beyond the keyword search parameters (i.e., Quantum AND Cryptograph*) used during the data collection phase, presenting a limitation. The reason why other similar search keywords (e.g., “Quantum AND Cryptanalysis”, “Quantum AND Data Security”) were not used was because such added keywords would have likely resulted in a significant increase in false positives. Fifth, the dataset is also limited with regard to the partial collection of data up until March 15, 2017. Sixth, a limitation exists with respect to *Scopus* being the sole source of data. Future studies can improve this dataset by collecting data from additional sources (e.g., *Web of Science*, *MathSciNet*). Lastly, bibliometric data are only, at best, an indirect measure to gauge the S&T research activities of a nation. External validity could have been improved with the inclusion of other types of data (e.g., patent, funding), but these were left out of this study due to practical limitations of time and resources. Thus, readers should use caution when generalizing the findings.

## Conclusions

Results from the first phase of analysis performed in this study show several QC research performance measures (PF, CS, H-index) comparing China with the other top five countries from 2001 to 2017. China’s PF dramatically increased from 2001 (i.e., 10) to 2016 (i.e., 196) with an Average Annual Growth Rate (AAGR) of 19.8% compared to CA’s 20.1%, DE’s 7.3%, US’s 6.9%, UK’s 3.4%, and the top five country median of 9.4% (see S2 File for AAGR formula). Though CA’s AAGR for PF is higher than China’s, CA had a low PF total from 2001 to 2017 compared to China (see S1 Table, CN: 1,477, CA: 436). China’s total CS was below that of the other top countries (see S1 Table), potentially indicating that China had less of an overall global impact on QC research. However, the AAGR for China’s CS (36.5%, see S1 Table) is considerably higher than that of its peers (i.e., US: 12.8%, UK: 10.4%, DE: 20.2%, CA: 18.3%), showing China’s exponentially increasing global impact. The H-index provided a normalized perspective illustrating that over the last decade China has taken the lead position in global QC research (see Fig 3). Moreover, the US’s steadily declining H-index growth (-11.9%, see S1 Table) provides an empirical indication that there has been a major shift in the international QC research landscape. It is still uncertain, however, how shifting from the US to China will impact the international order in cyberspace, since QC technologies have not been fully realized. China’s successful QC research performance might be a product of its government’s education and economic strategy. According to the US National Science Board, China has outpaced the European Union and the US in terms of the amount of first science and engineering university degrees granted to citizens [63]. In addition, China was also found to have the “most vigorous growth” in percentage of Gross Domestic Product spend on R&D [63].

Previous policy research provided subjective speculation on China’s quantum S&T research advances based on a review of several Chinese research and policy papers [1,2], whereas results from the second phase of analysis in this study used computational techniques to quantitatively analyze, in an objective fashion, a large set of data (co-words) extracted from China’s QC research publications at a scale beyond that which could reasonably be accomplished with a manual review. The thematic structure of China’s QC research visually mapped the domain’s development over four time periods (i.e., 2001–2004, 2005–2008, 2009–2012, 2013–2017). QC and S&T policy researchers can use these maps to trace previous research directions and plan future lines of research in order to align the domain with strategic objectives. For example, several themes were observed to carry over between time periods (e.g., PHOTONS, QUANTUM-KEY-DISTRIBUTION, SECRET-MESSAGES, QUANTUM-OPTICS, QUANTUM-SIGNATURES), and some themes disappeared with time (e.g., COMPUTER-NETWORKS, ATTACK-STRATEGIES, BELL-STATE, POLARIZATION-STATE), while others emerged more recently (e.g., QUANTUM-ENTANGLEMENT, DECOY-STATE, UNITARY-OPERATION). Themes also underwent analysis based on performance measures to identify those themes that have had the most global research impact (e.g., QUANTUM-KEY-DISTRIBUTION, PHOTON-OPTICAL-COMMUNICATION, NETWORK-PROTOCOLS and QUANTUM-ENTANGLEMENT). These findings illustrate the utility of using computational and quantitative methods to efficiently and effectively map the development of a research domain. These findings support S&T policy researchers in gaining better insight into the outcome of investments. QC researchers can also use this study to objectively plot where their domain is headed. Considering China is the premier driving force in global QC research, an understanding of China’s QC research themes helps provide clarity into how QC technologies will impact the cyber domain. China has transitioned basic research themes such as QUANTUM-ENTANGLEMENT to applied research practice by generating the first successful quantum teleportation between separated macroscopic objects, which moved China further towards QC secured communication networks [64].

## Supporting information

S1 File Cluster keyword networks. (PDF)

S2 File Calculating the Annual Average Growth Rate (AAGR) for performance measures. (PDF)

S1 Table Summarizing table with total performance measures from 2001–2017 along with their Annual Average Growth Rate (AAGR) from 2001–2016 for the top five quantum cryptography research countries. (PDF)

S2 Table Publication Frequency table for top five quantum cryptography research countries from 2001–2017. (PDF)

S3 Table Citation Score table for top five quantum cryptography research countries from 2001–2017. (PDF)

S4 Table H-index table for top five quantum cryptography research countries from 2001–2017. (PDF)

S5 Table China’s Collaboration Frequency for quantum cryptography research from 2001–2017. (PDF)
