# Supplementary material for: Examination of China’s performance and thematic evolution in quantum cryptography research using quantitative and computational techniques
Source: PLoS One. 2018 Jan 31;13(1):e0190646. doi: 10.1371/journal.pone.0190646 (PMC5791966; doi:10.1371/journal.pone.0190646)

S1 File. Cluster keyword networks.

2001-2004

OPTICAL-COMMUNICATION

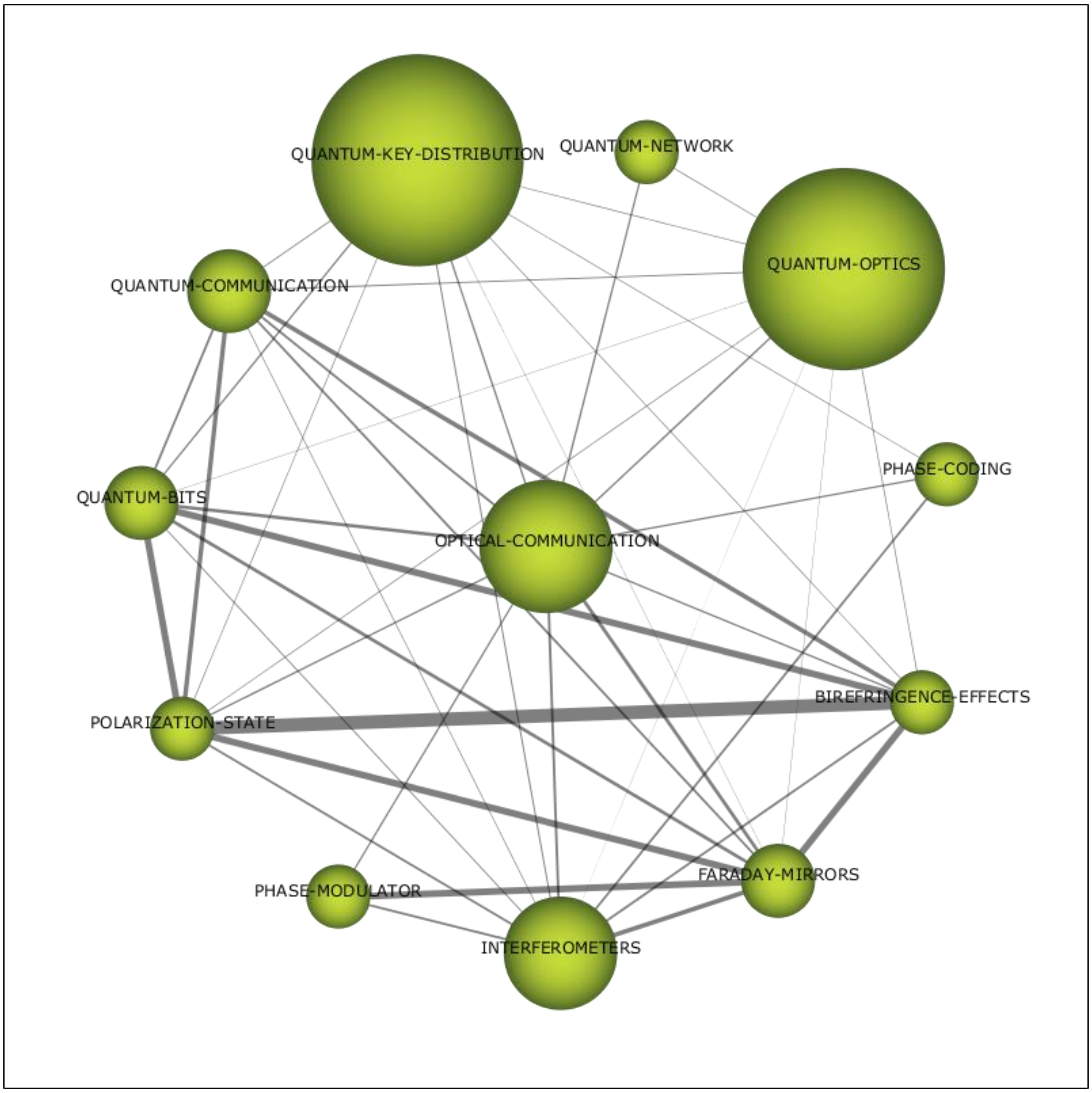

ALGORITHMS

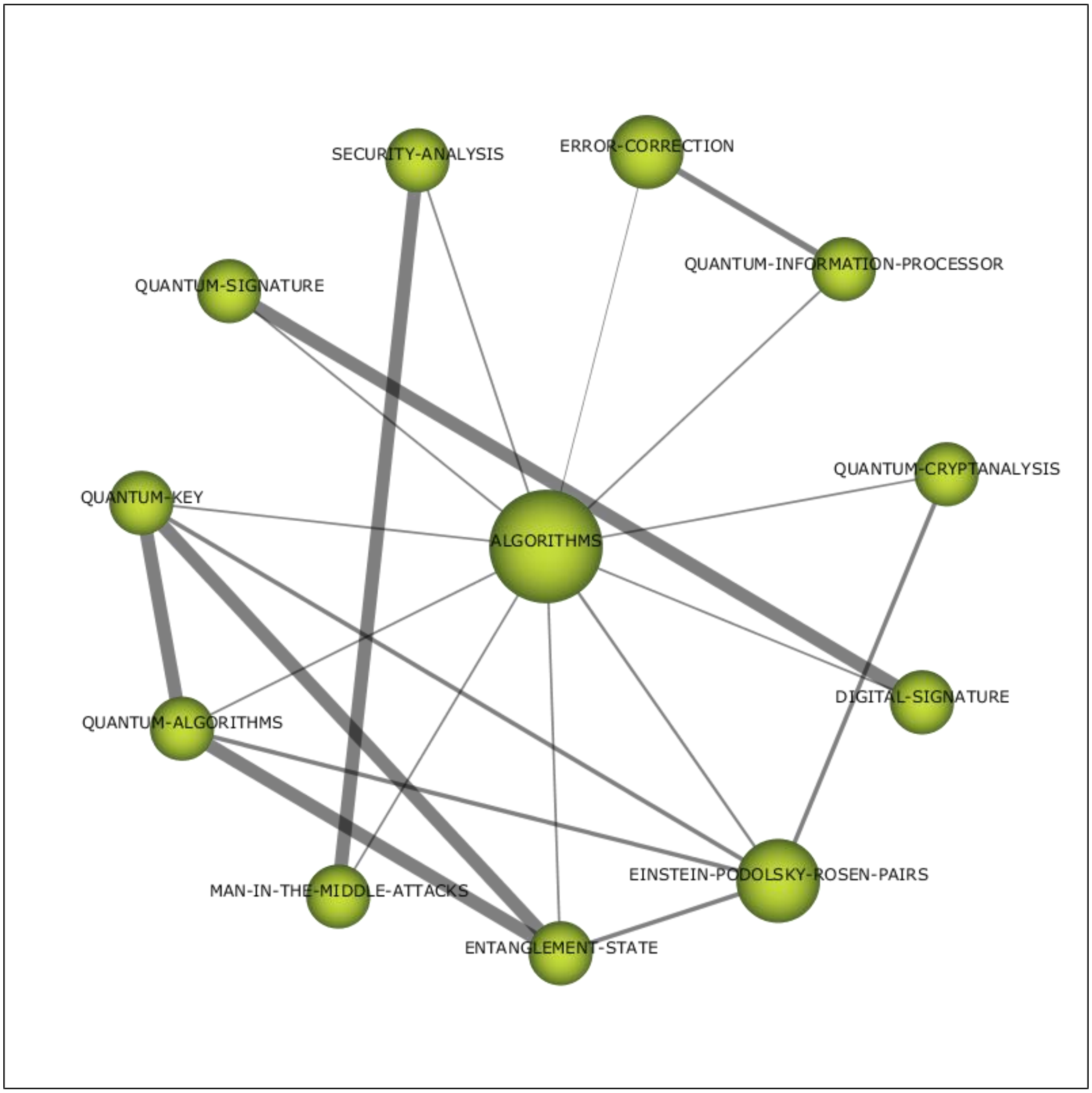
SECURITY-ANALYSIS
ERROR-CORRECTION
QUANTUM-SIGNATURE
QUANTUM-INFORMATION-PROCESSOR
QUANTUM-CRYPTANALYSIS
QUANTUM-KEY
ALGORITHMS
DIGITAL-SIGNATURE
QUANTUM-ALGORITHMS
EINSTEIN-PODOLSKY-ROSEN-PAIRS
MAN-IN-THE-MIDDLE-ATTACKS
ENTANGLEMENT-STATE

QUANTUM-KEY-DISTRIBUTION-(QKD)

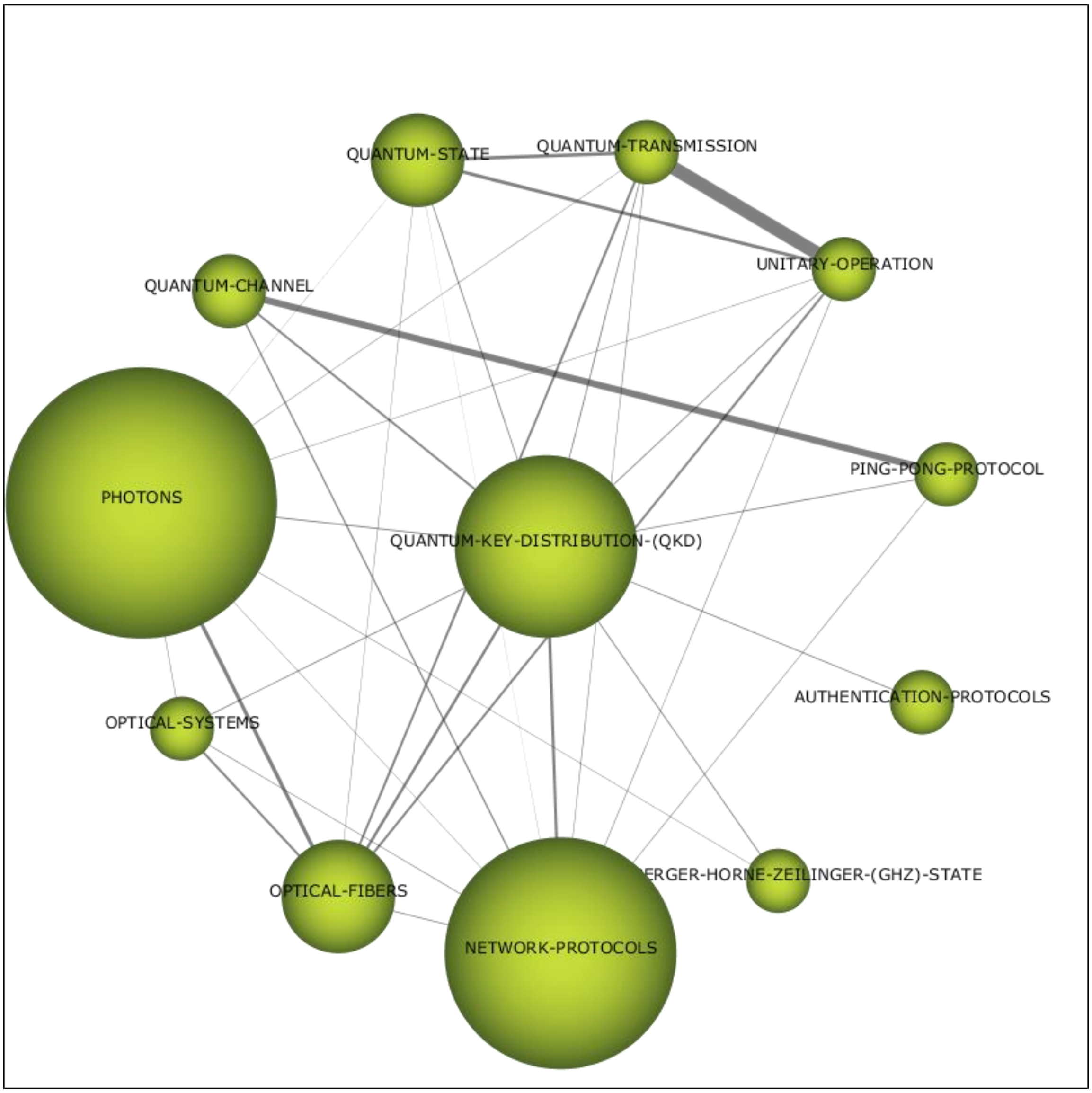

BELL-STATE

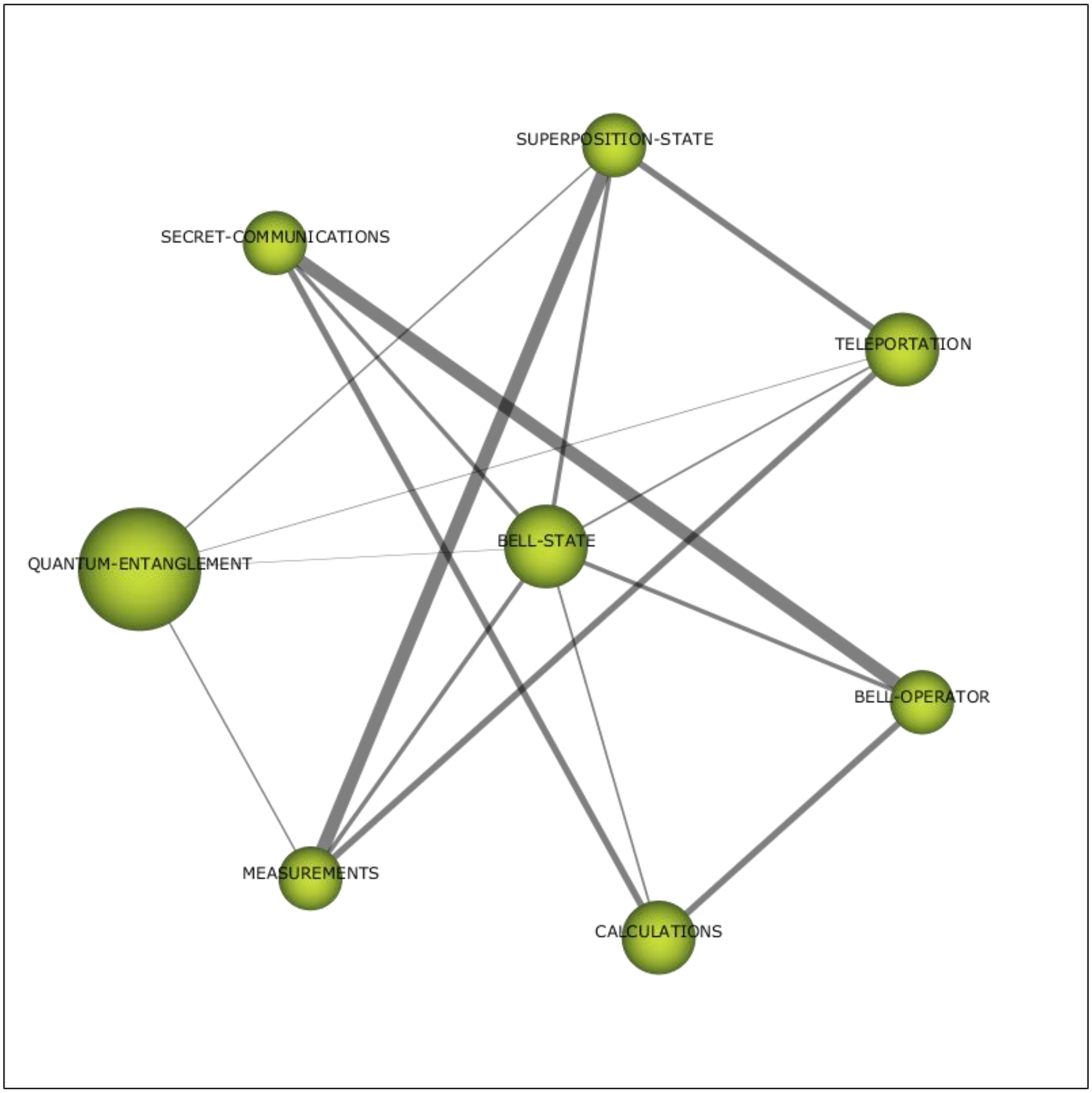
SUPERPOSITION-STATE
SECRET-COMMUNICATIONS
TELEPORTATION
QUANTUM-ENTANGLEMENT
BELL-STATE
BELL-OPERATOR
MEASUREMENTS
CALCULATIONS

PUBLIC-KEY-CRYPTOGRAPHY

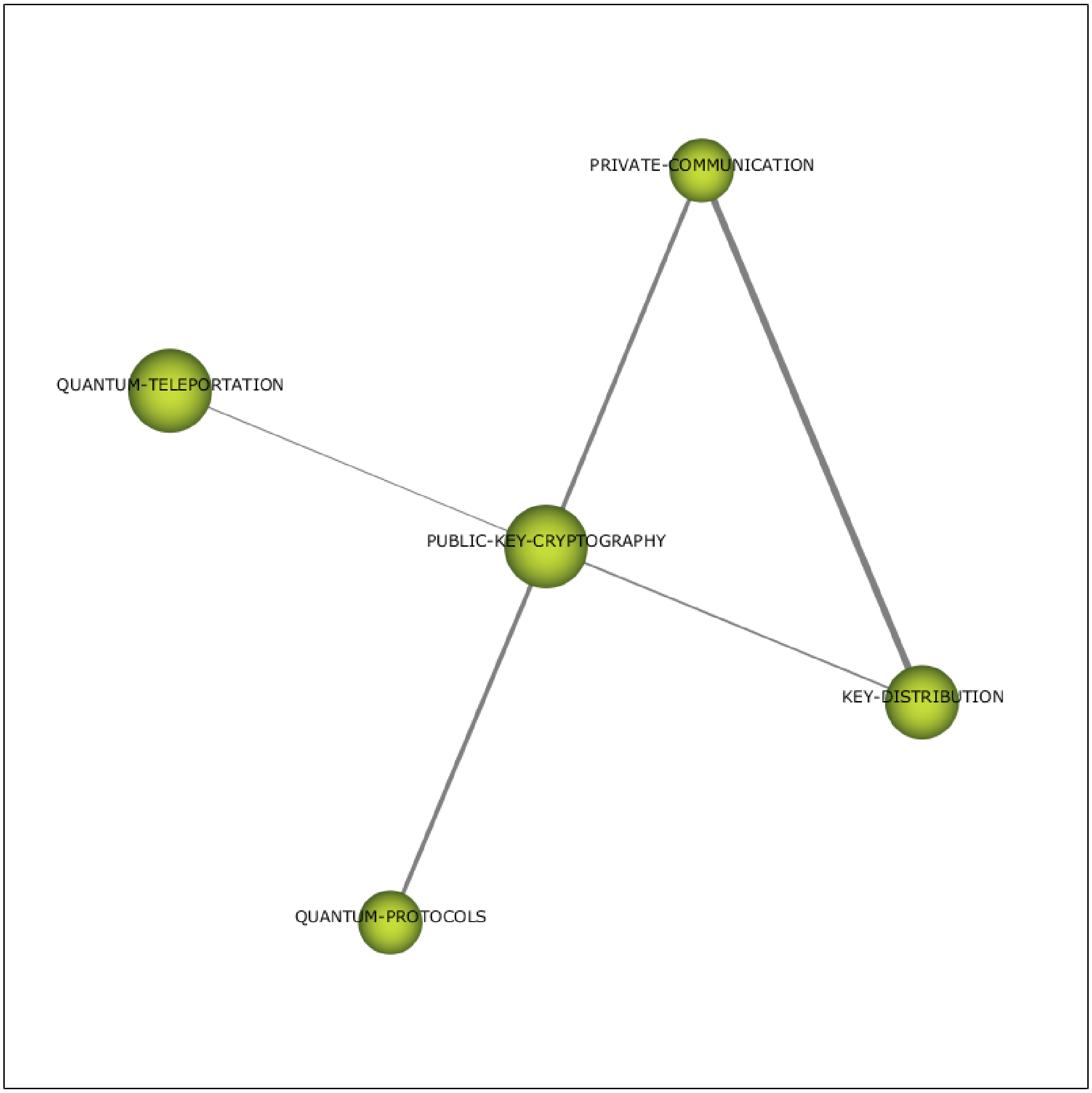

BB84-PROTOCOL

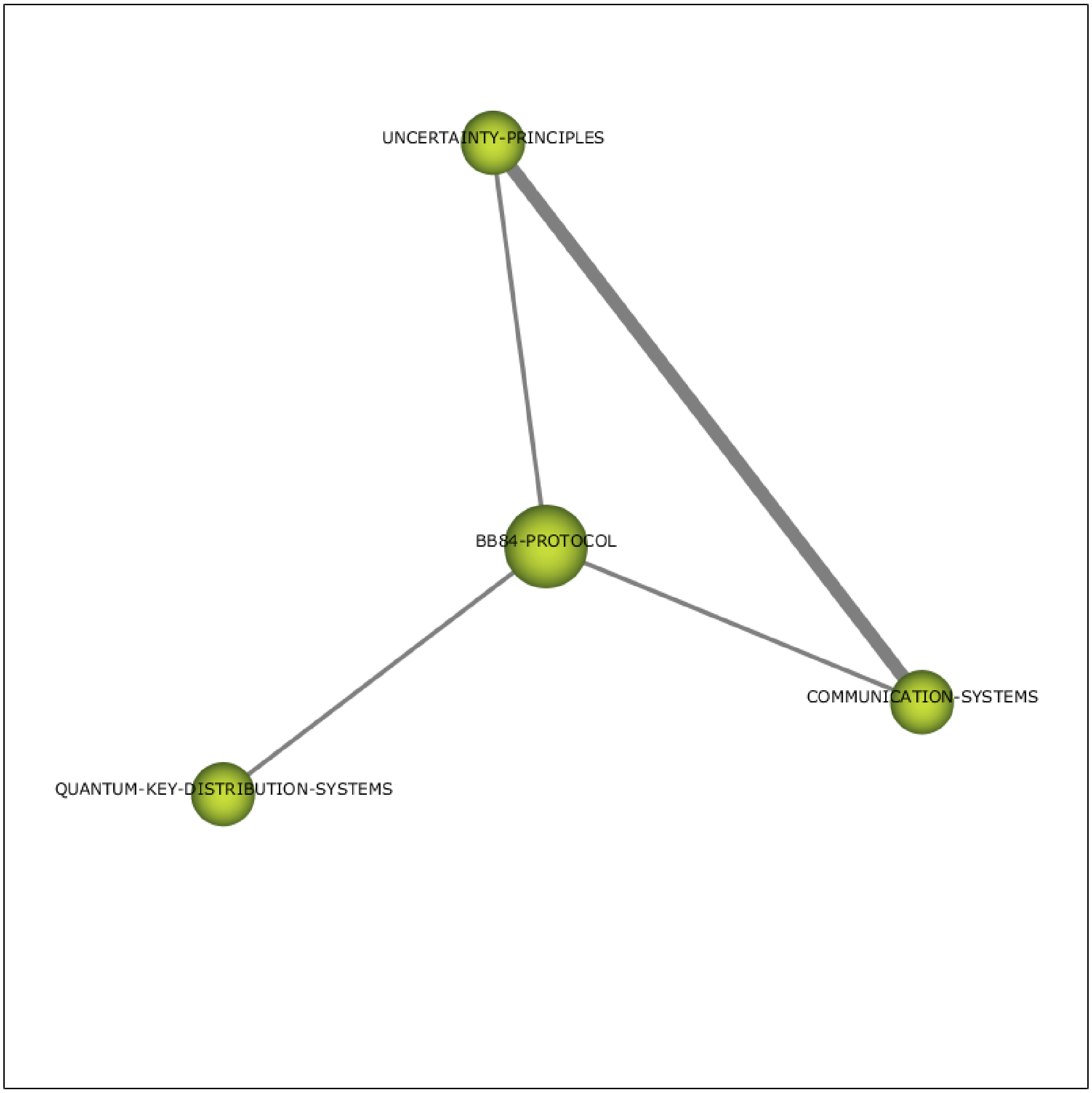

COMPUTER-NETWORKS

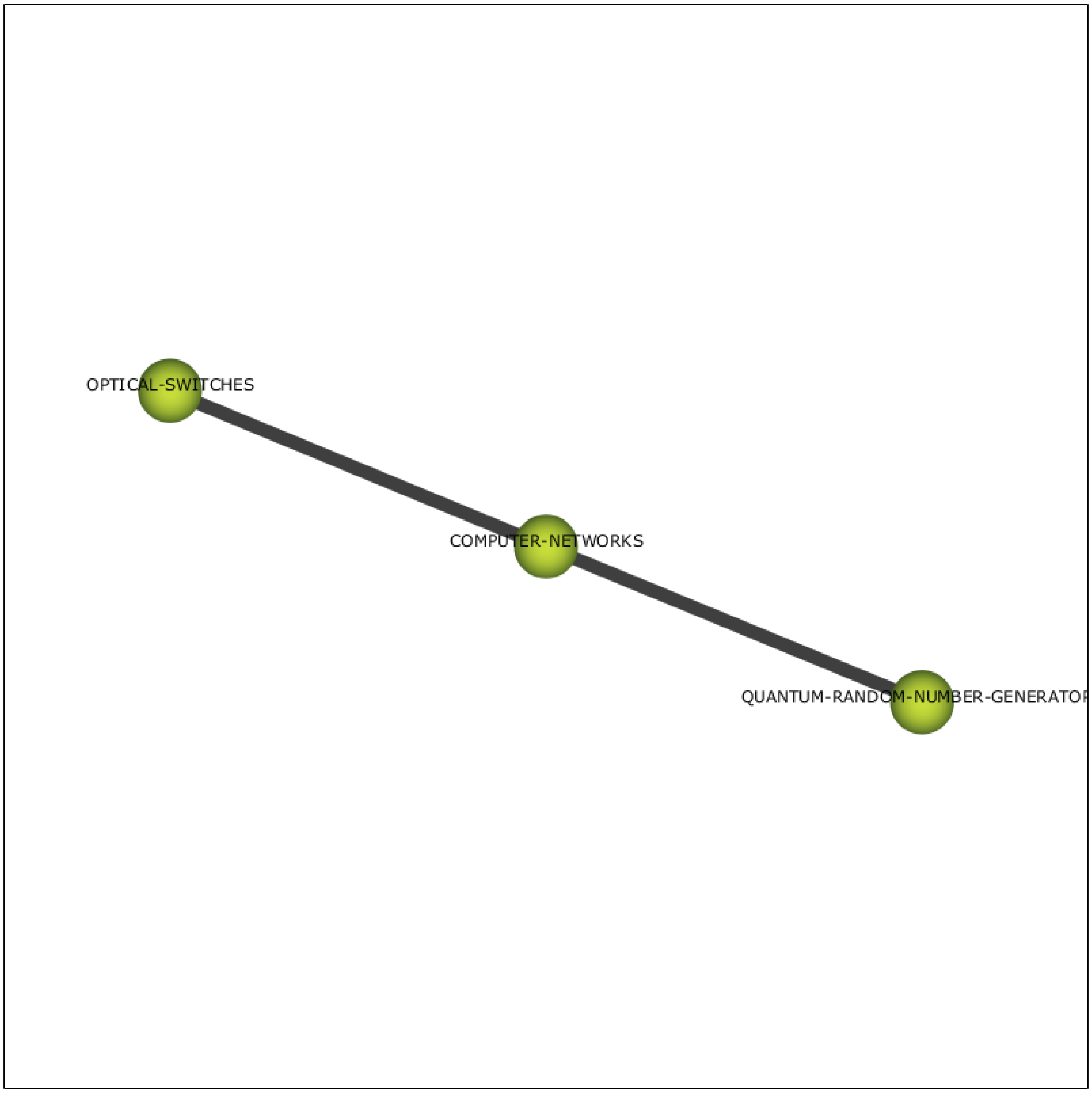

ATTACK-STRATEGIES

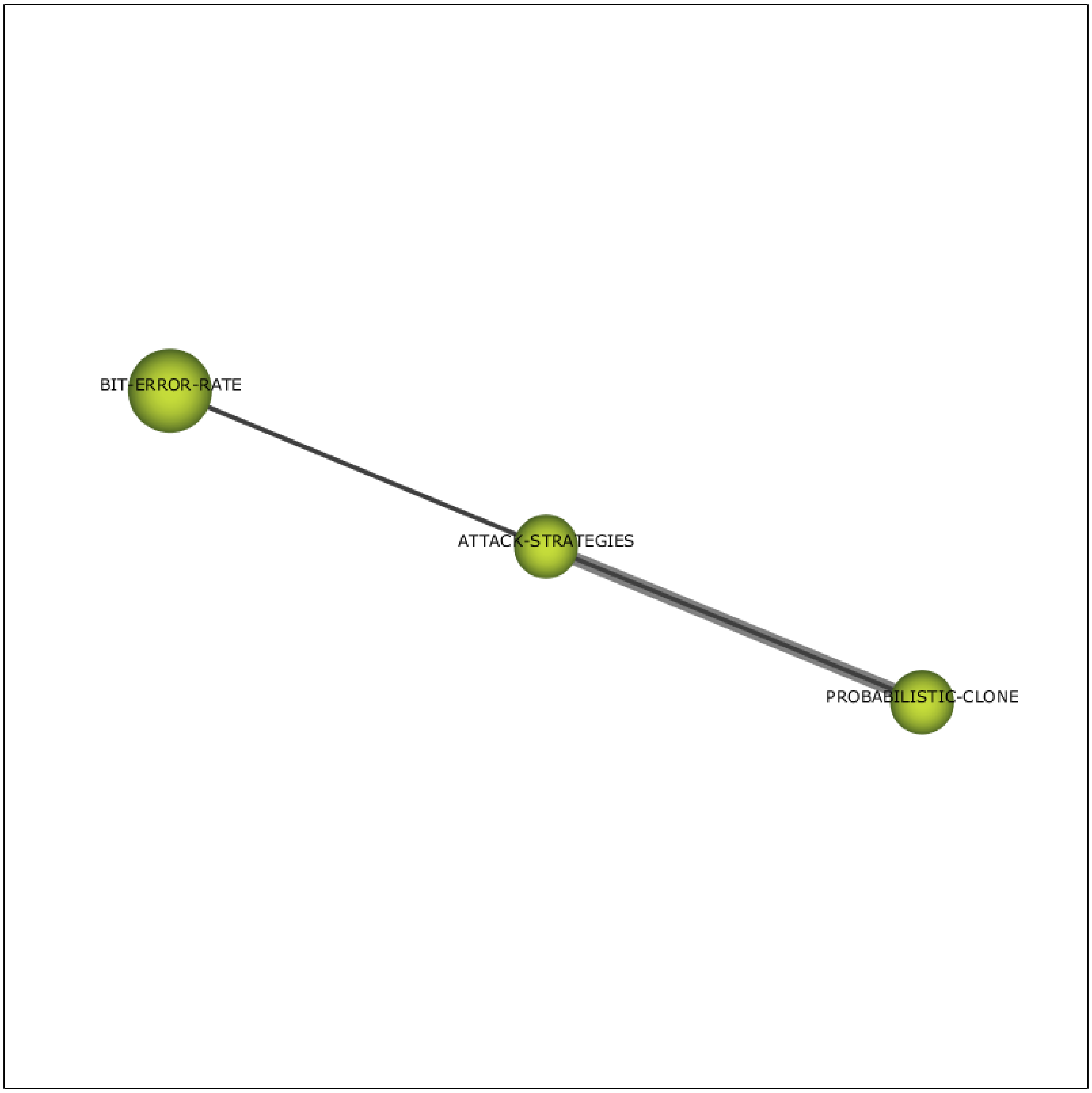

2005-2008

PHOTONS

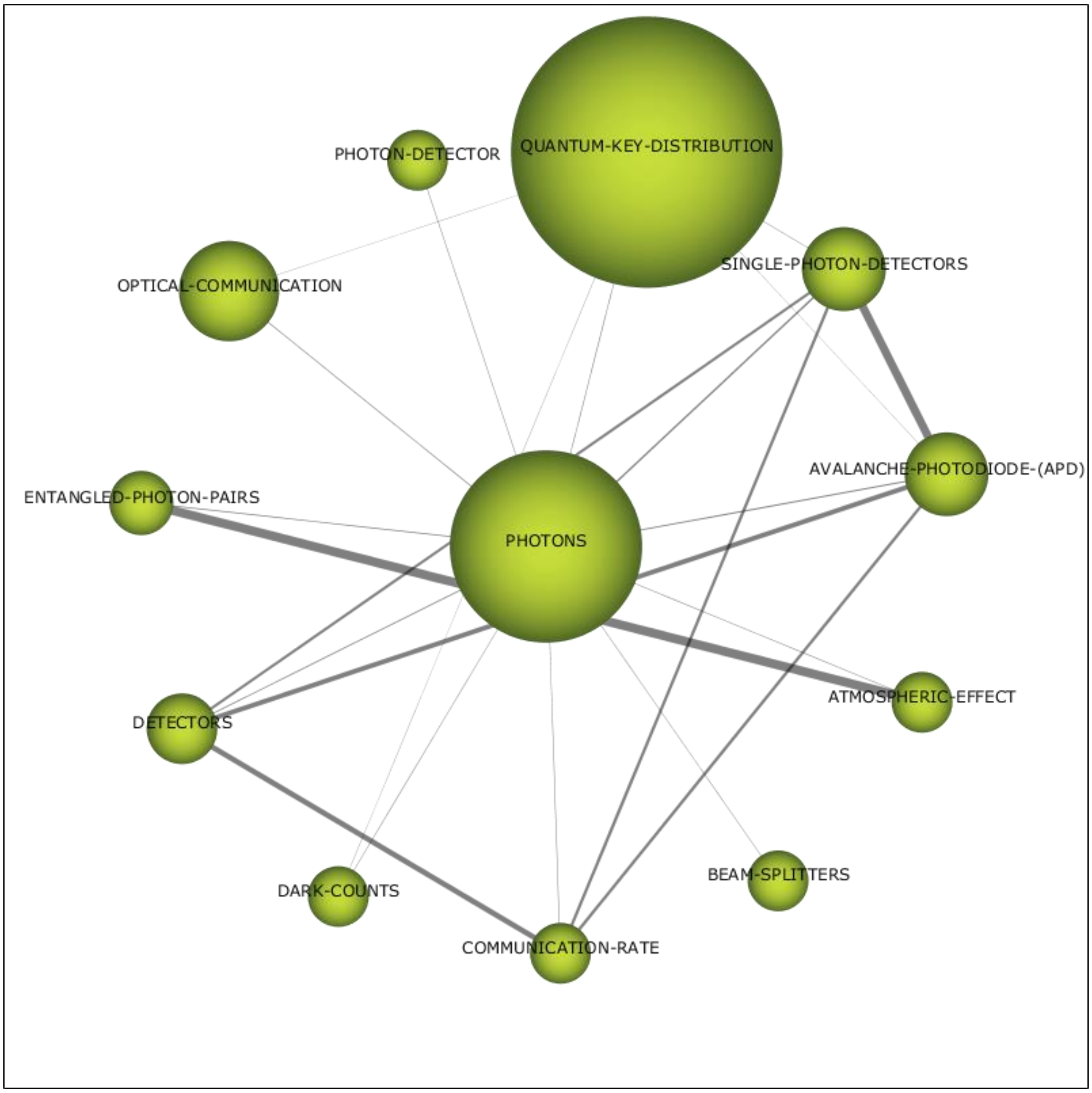

NETWORK-PROTOCOLS

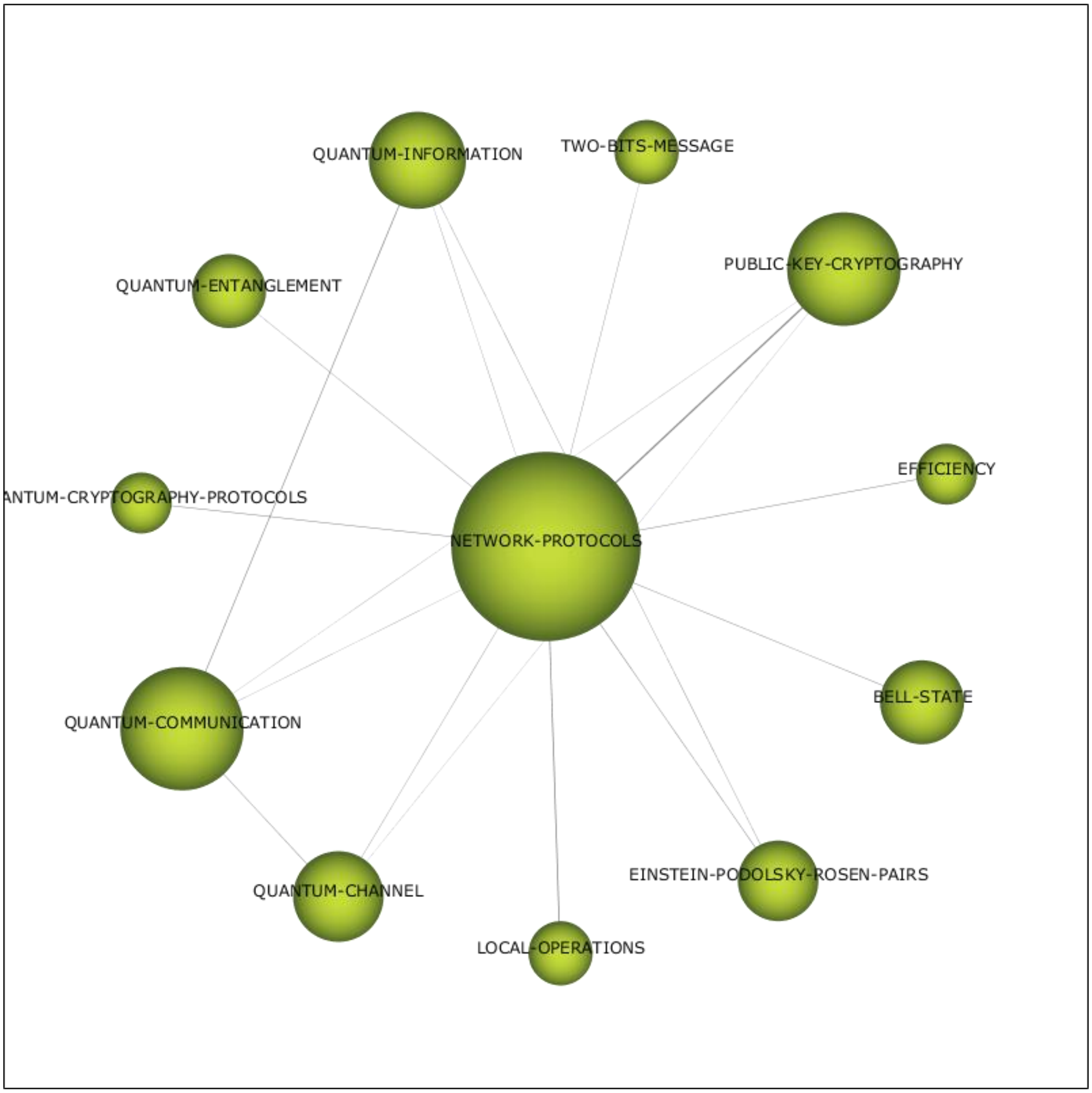

SECRET-MESSAGES

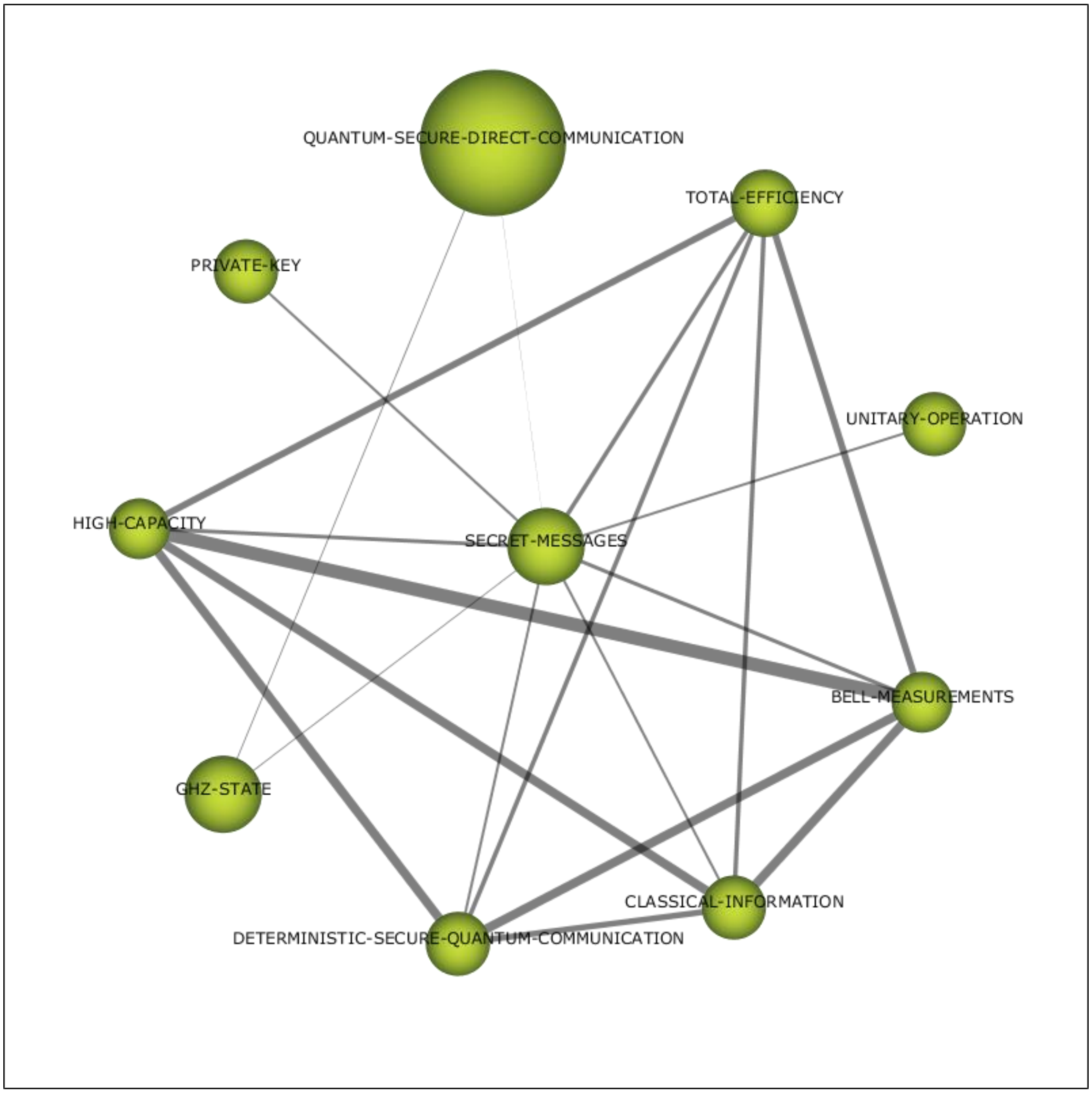

QUANTUM-OPTICS

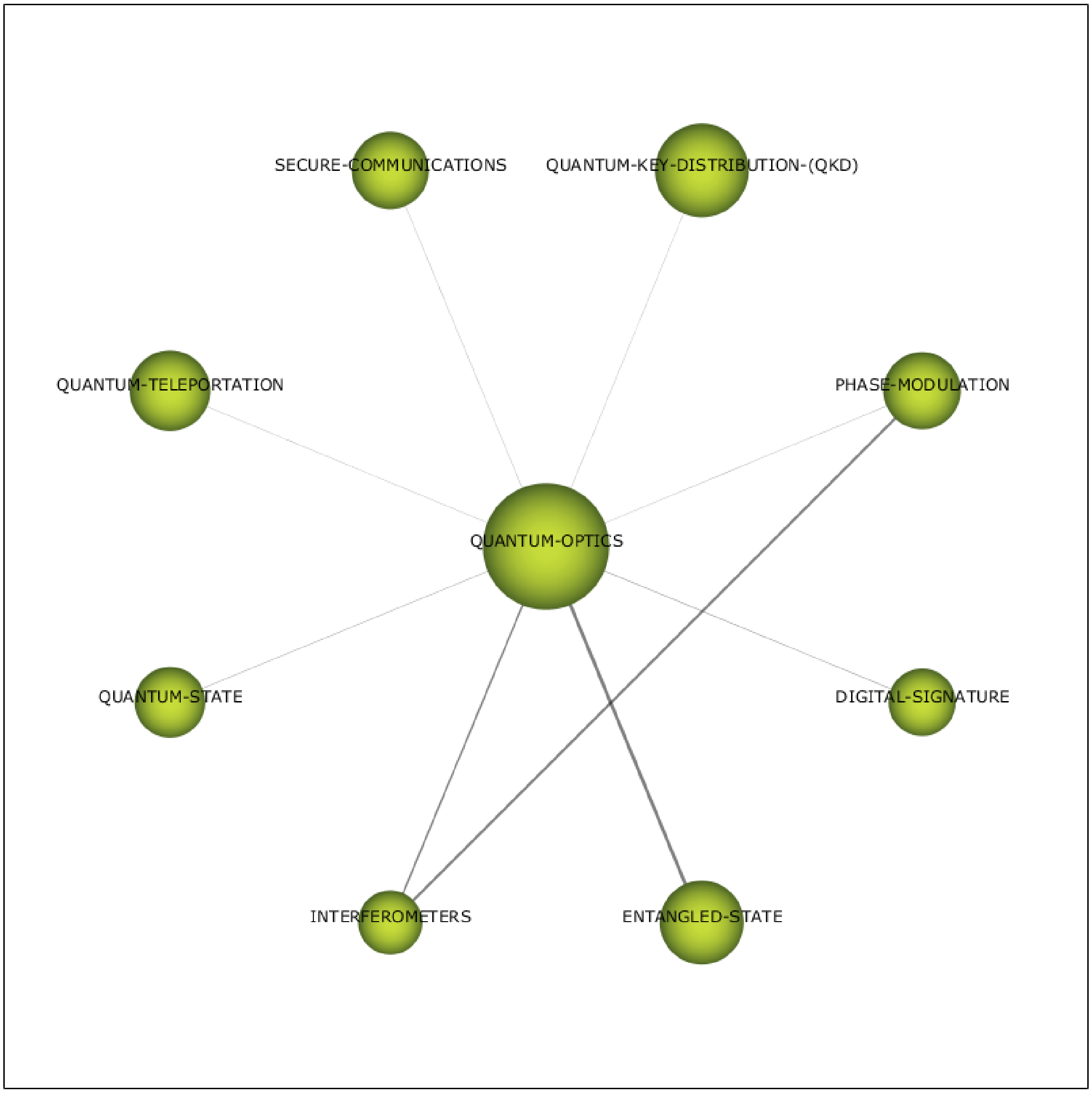

POLARIZATION-STATE

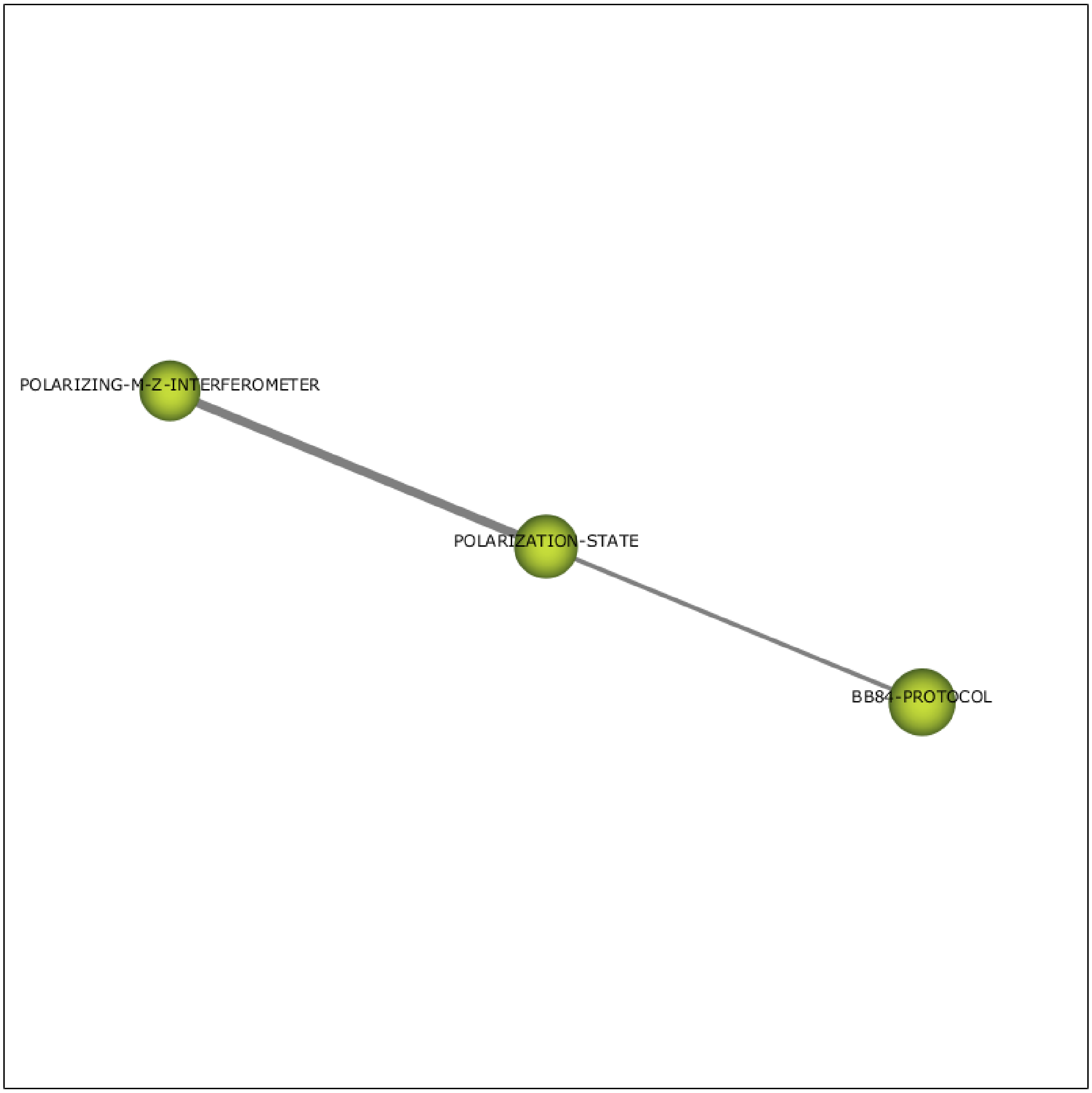

ALGORITHMS

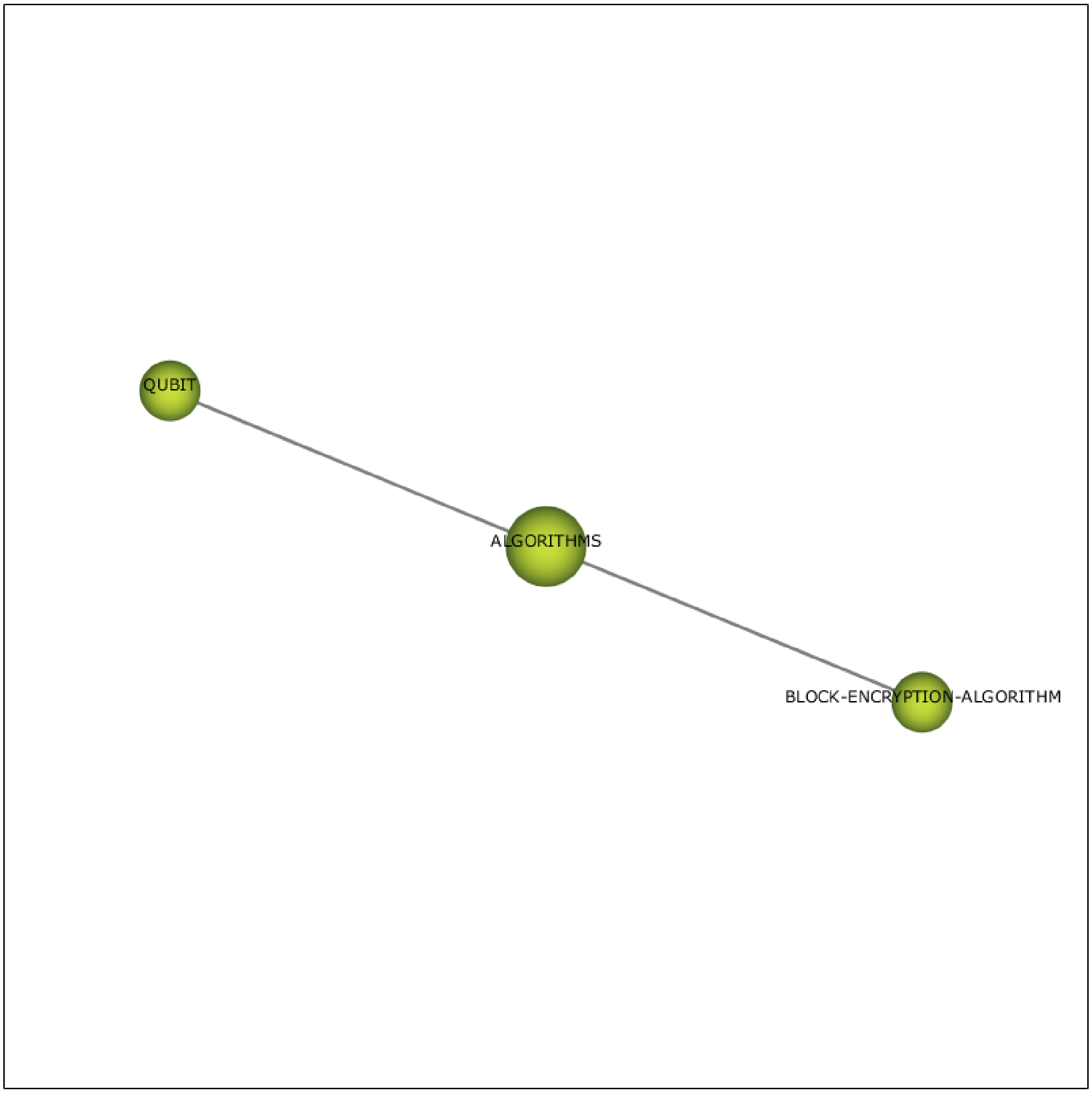

OPTICAL-FIBERS

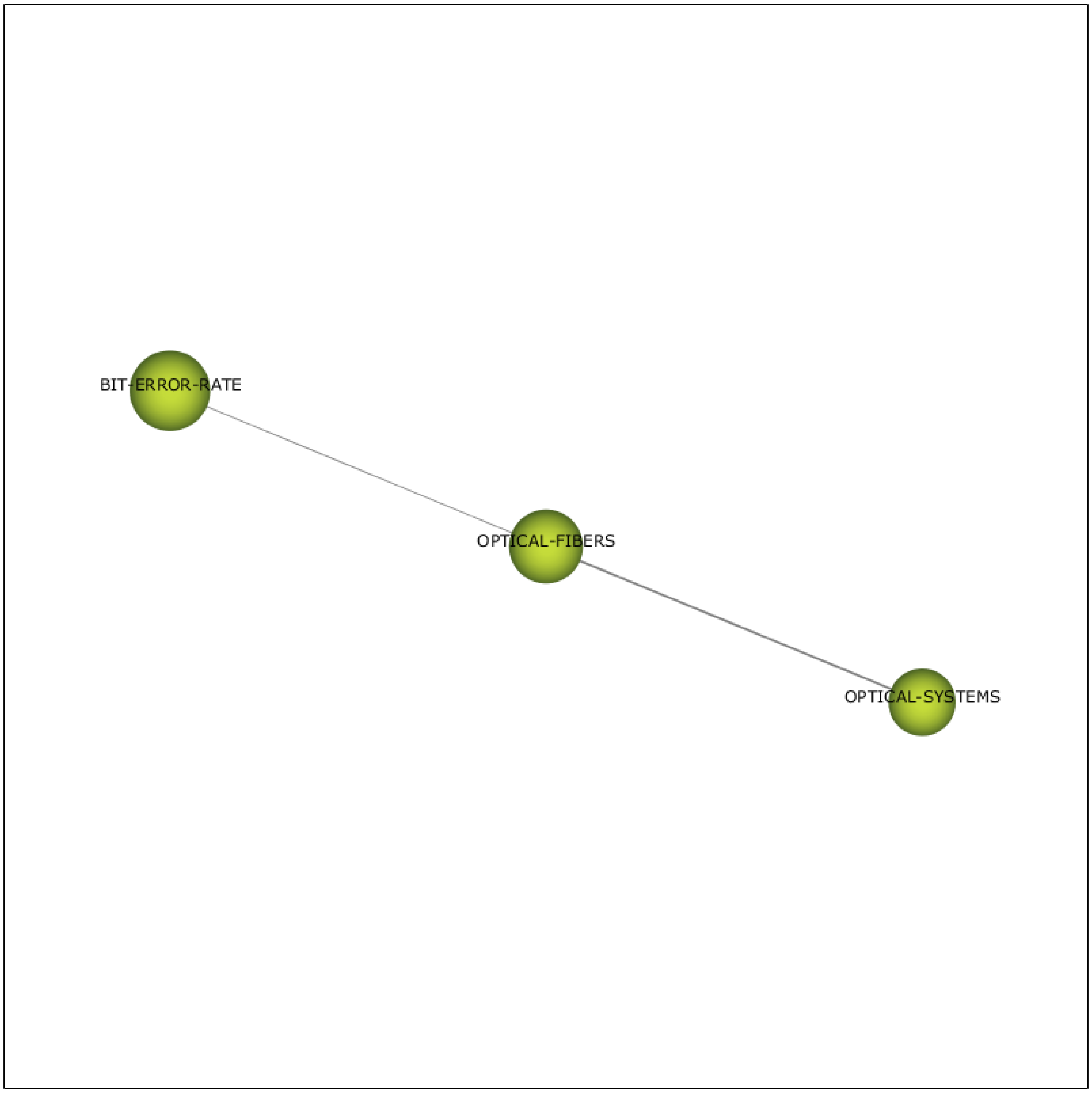

2009-2012

PHOTONS

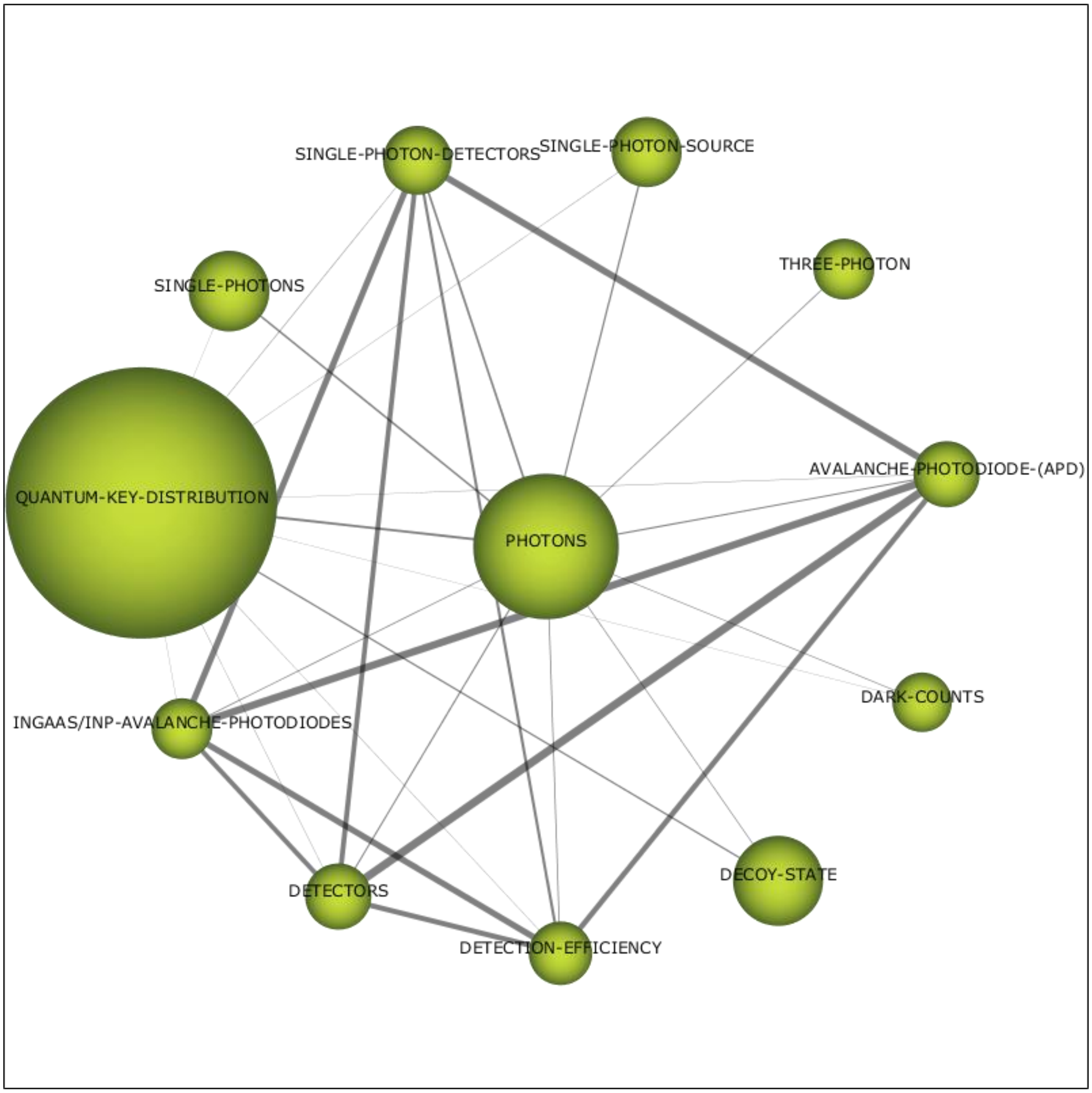

SECRET-MESSAGES

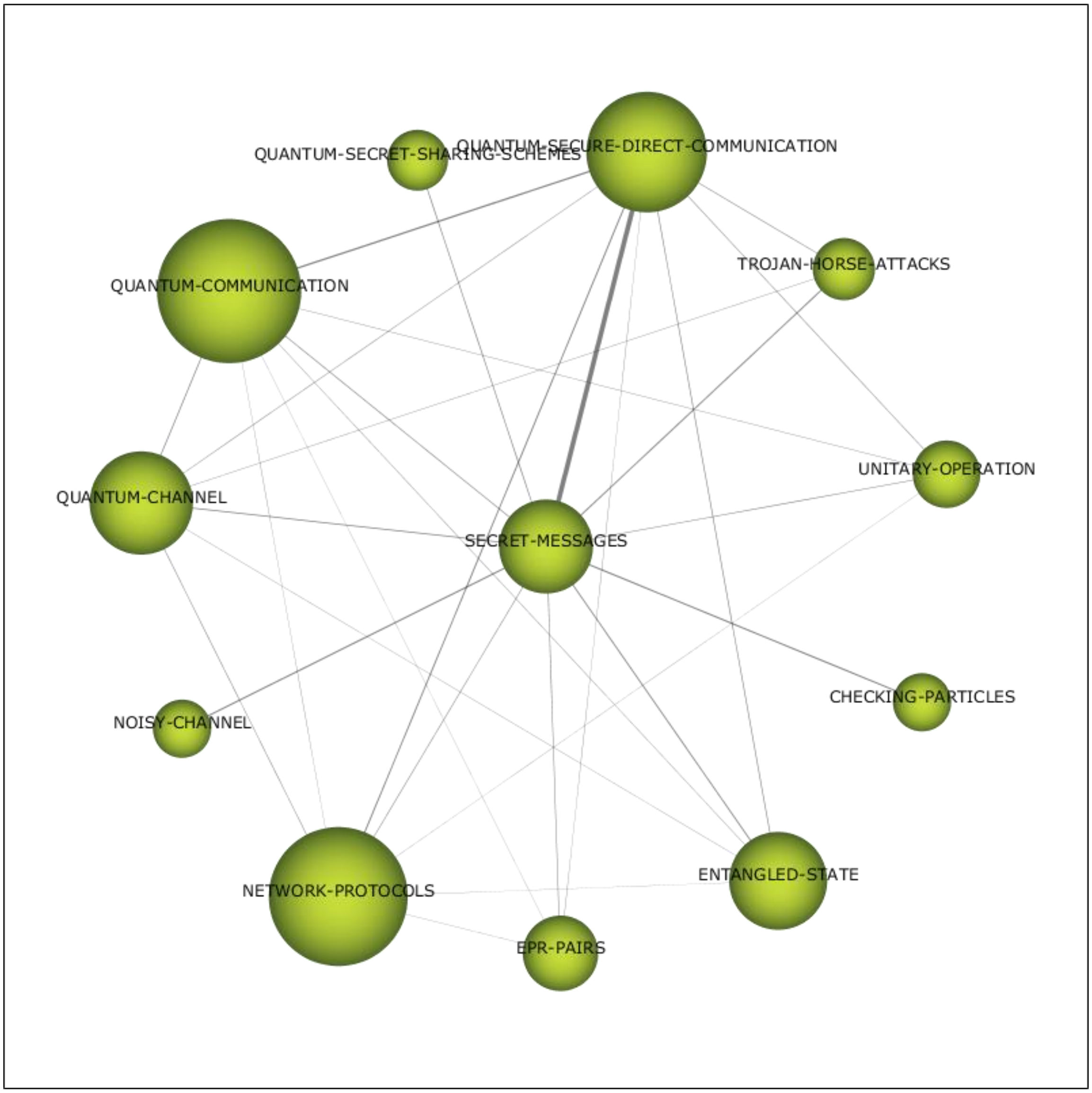

QUANTUM-OPTICS

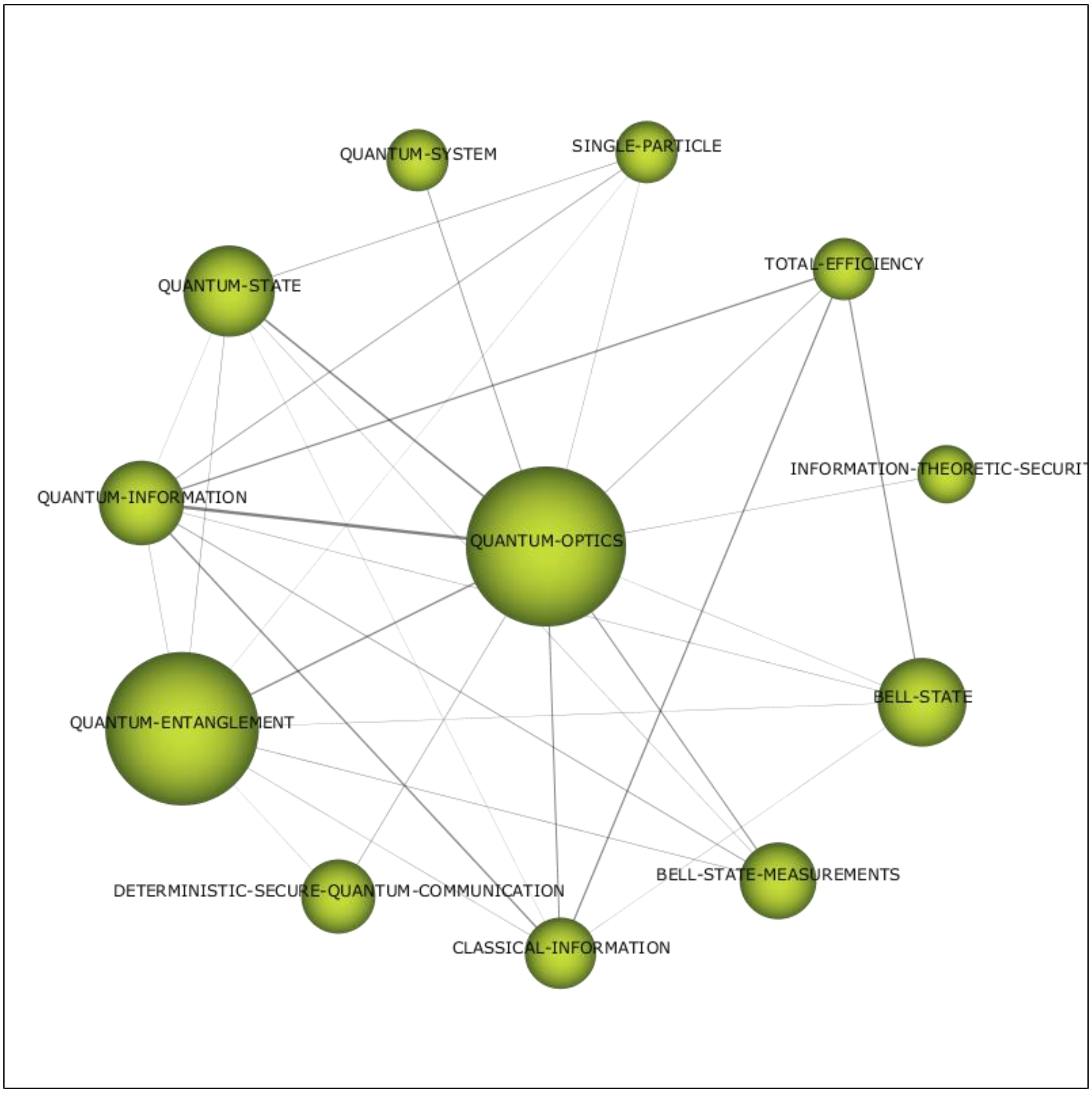

QUANTUM-KEY-DISTRIBUTION-PROTOCOLS

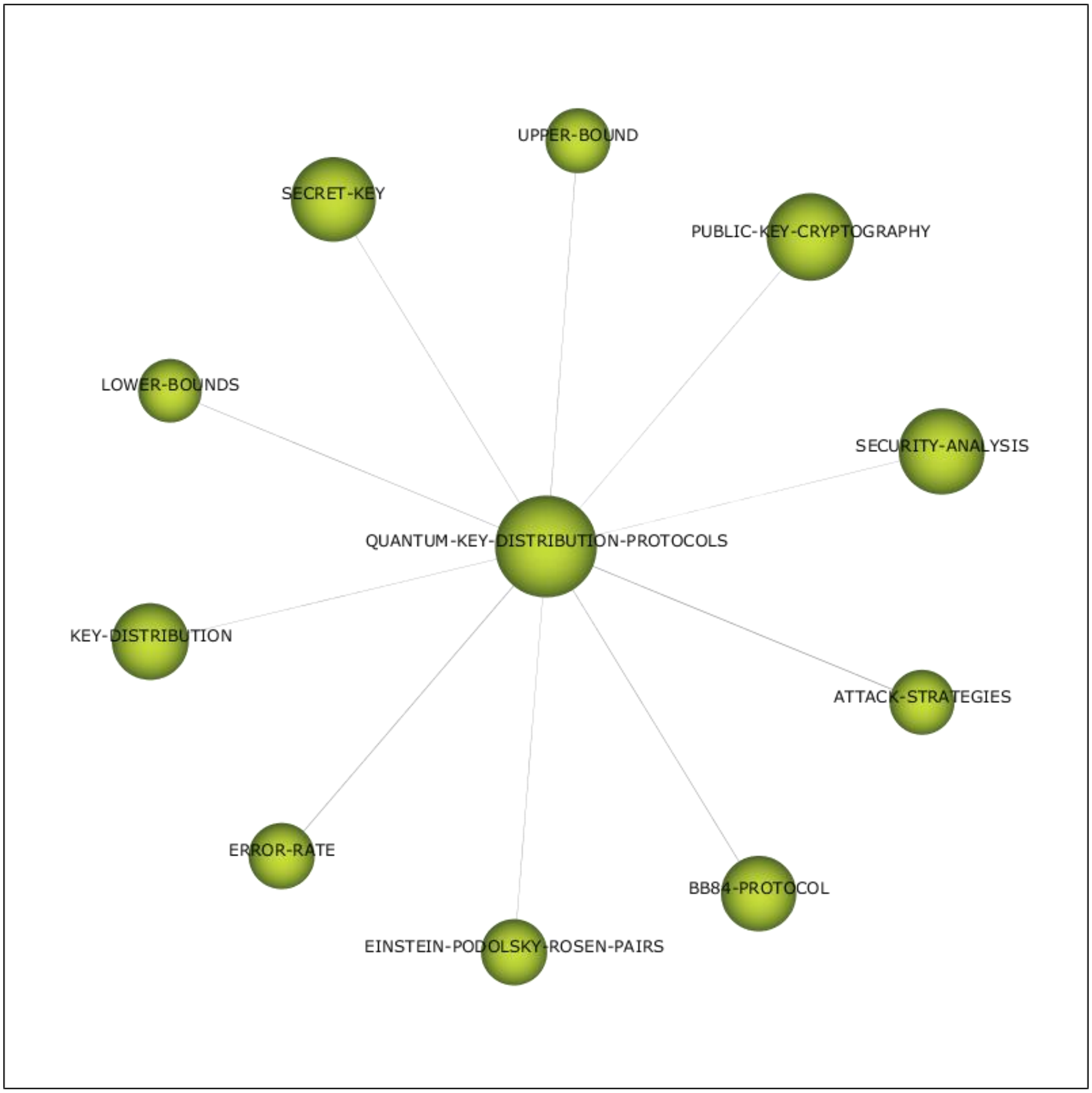

OPTICAL-COMMUNICATION

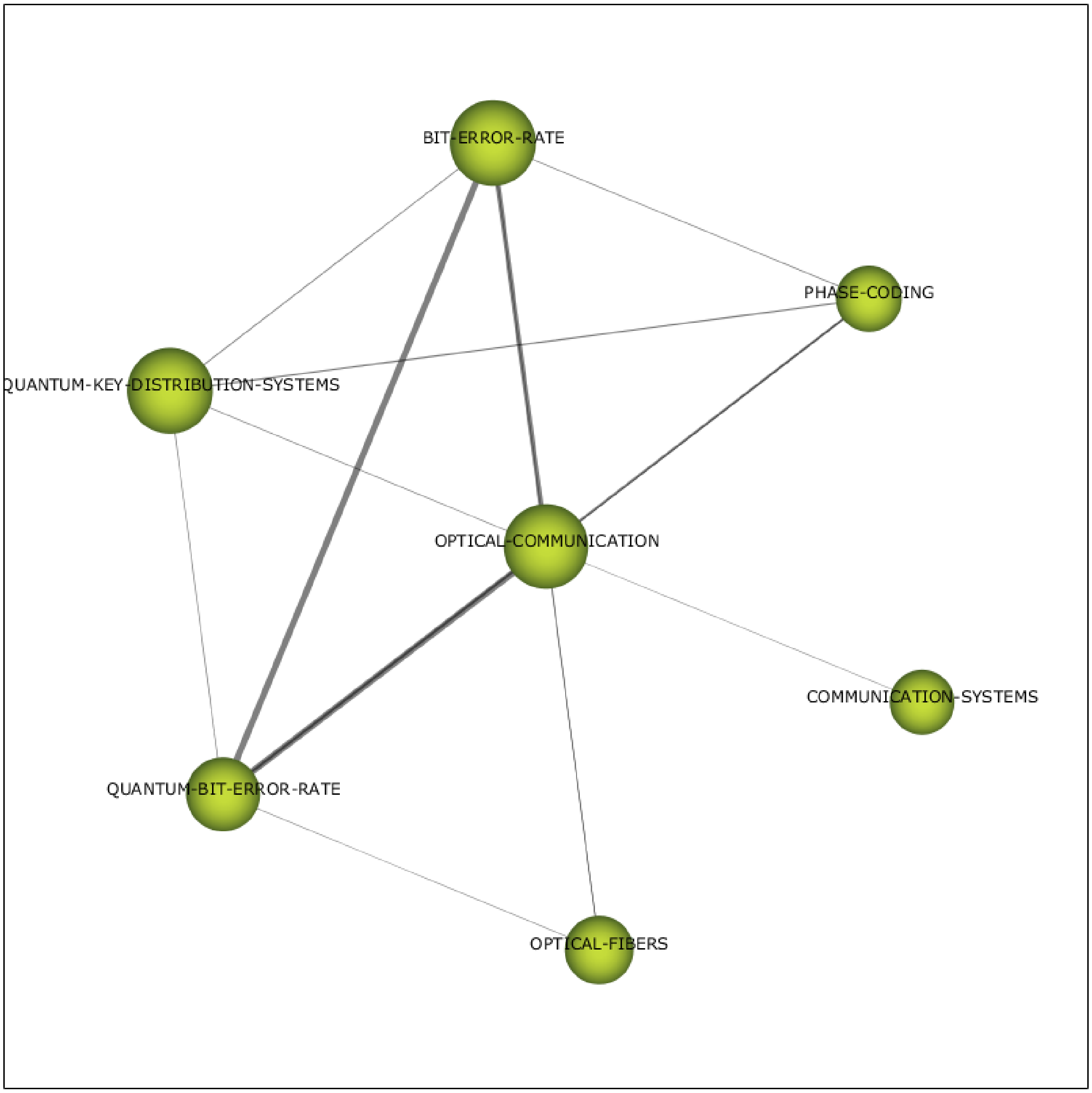

QUANTUM-SIGNATURE

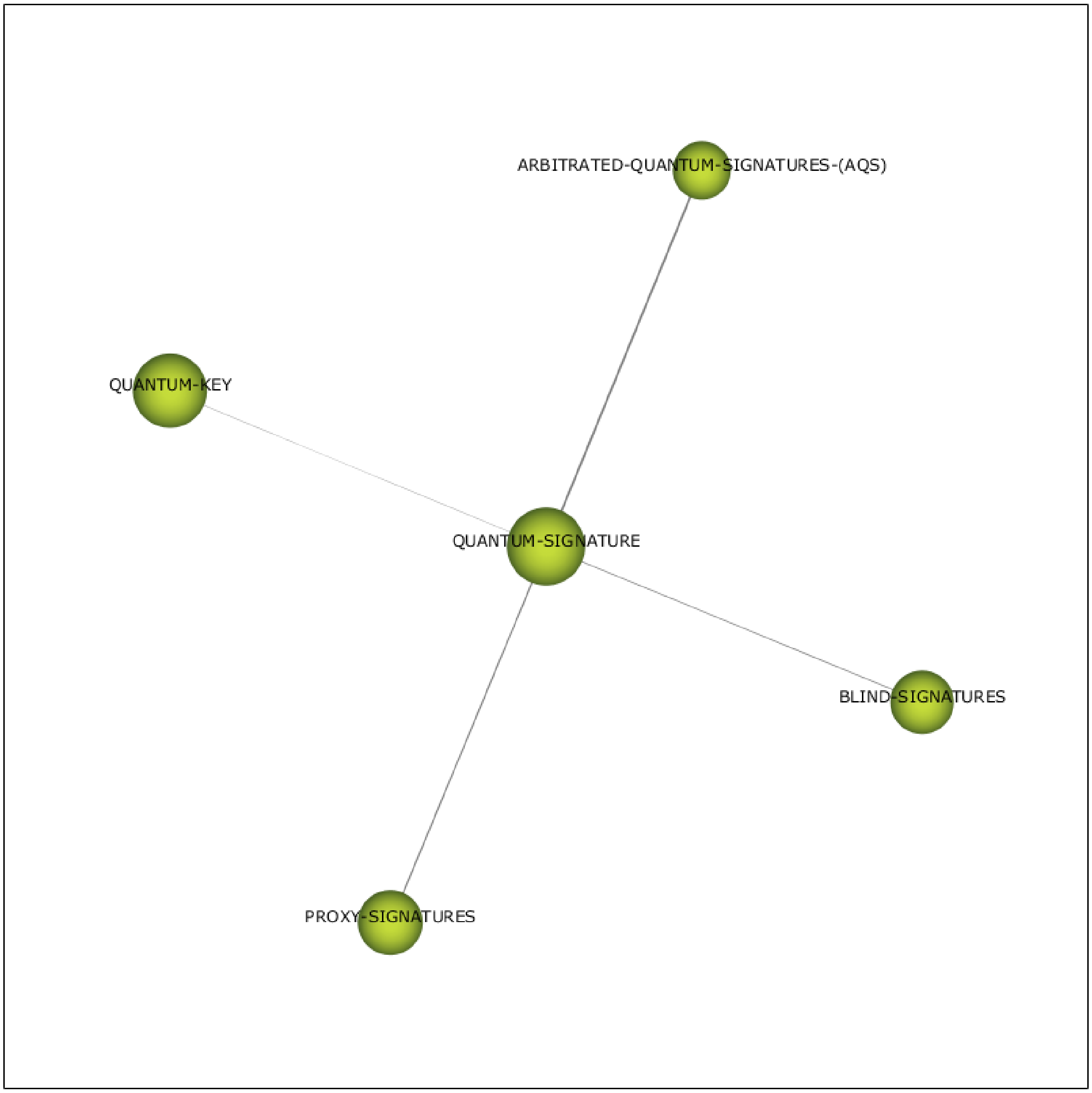

QUANTUM-SECURE-COMMUNICATION

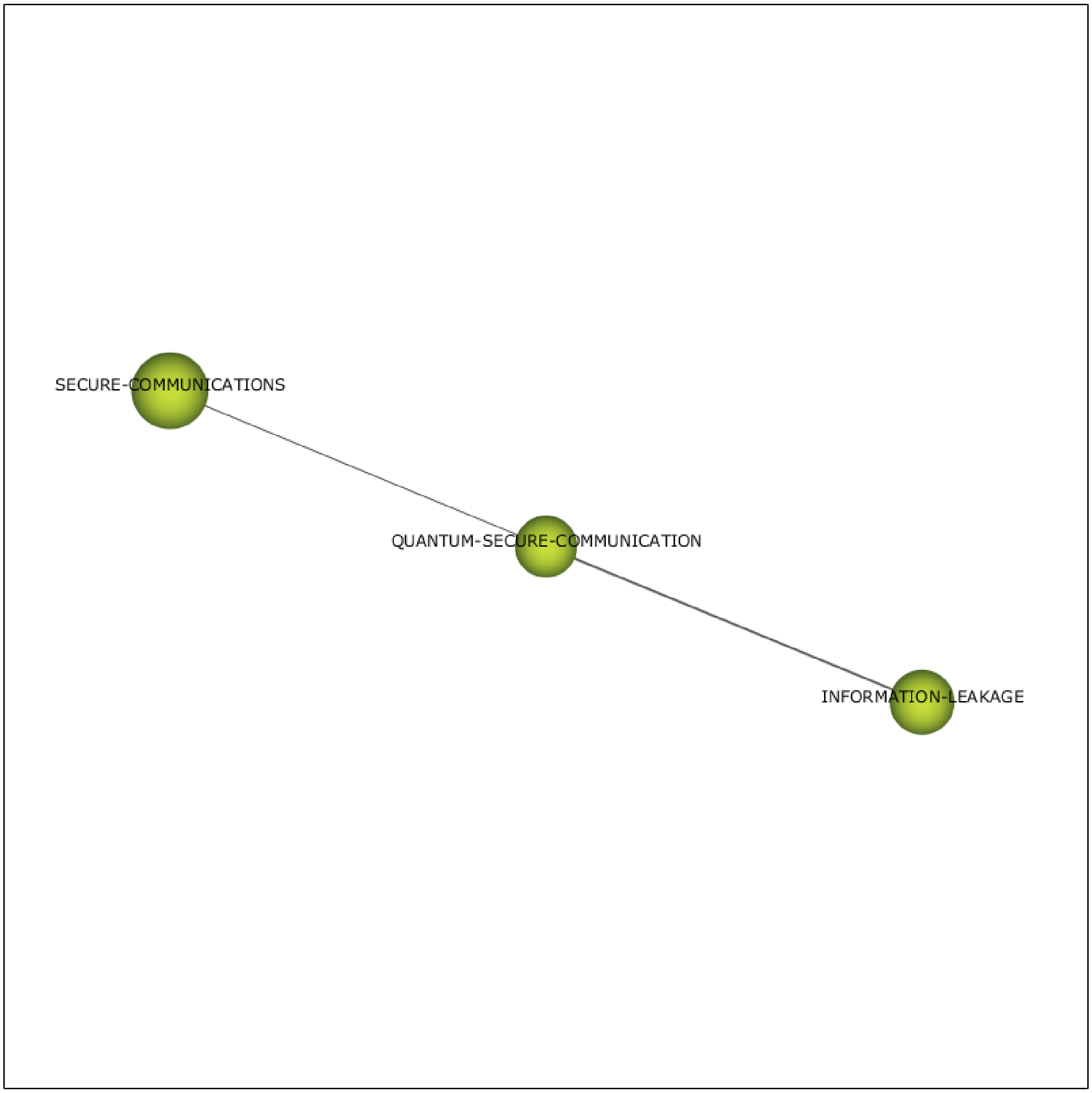

2013-2017

PHOTONS

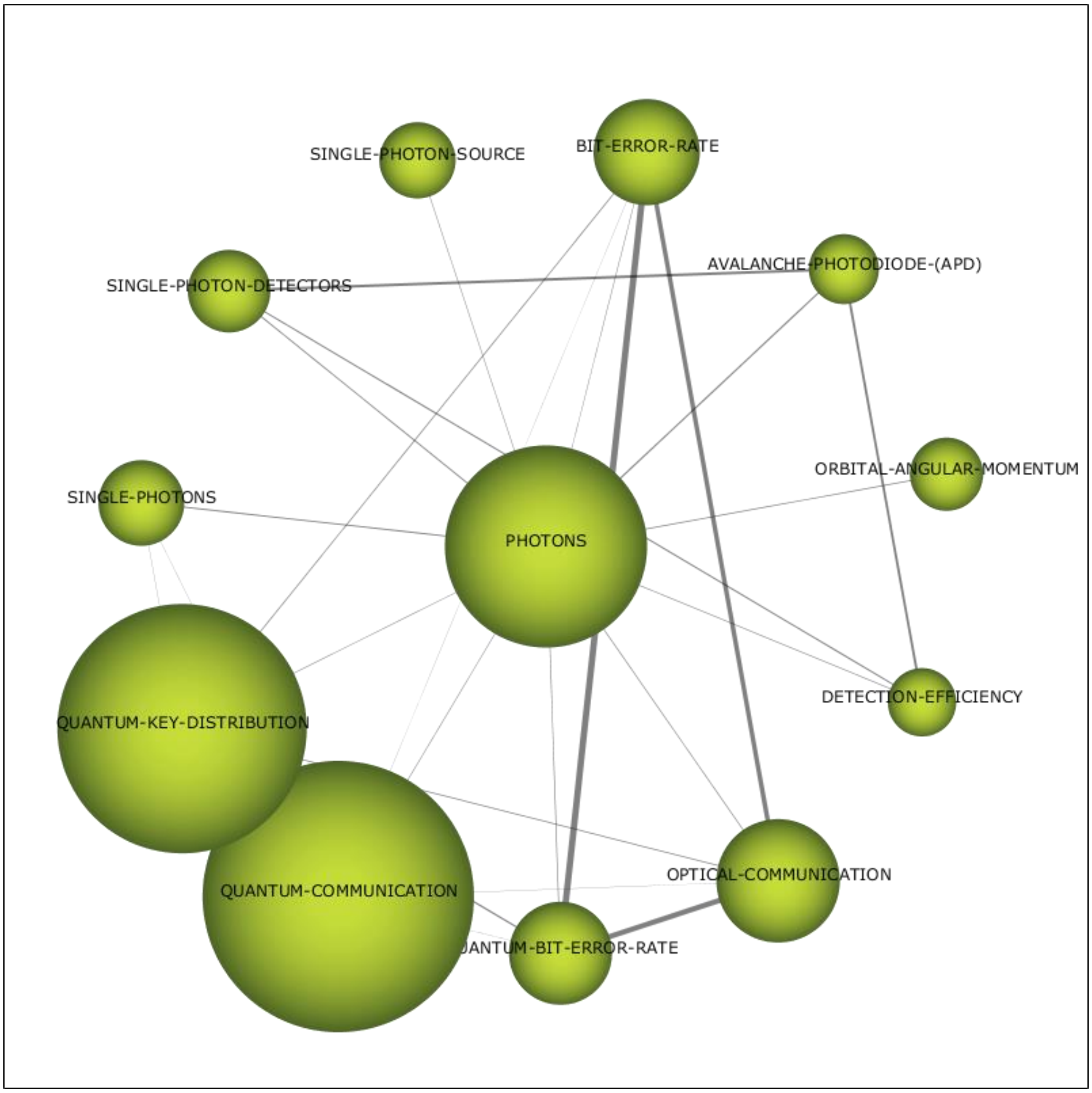

QUANTUM-ENTANGLEMENT

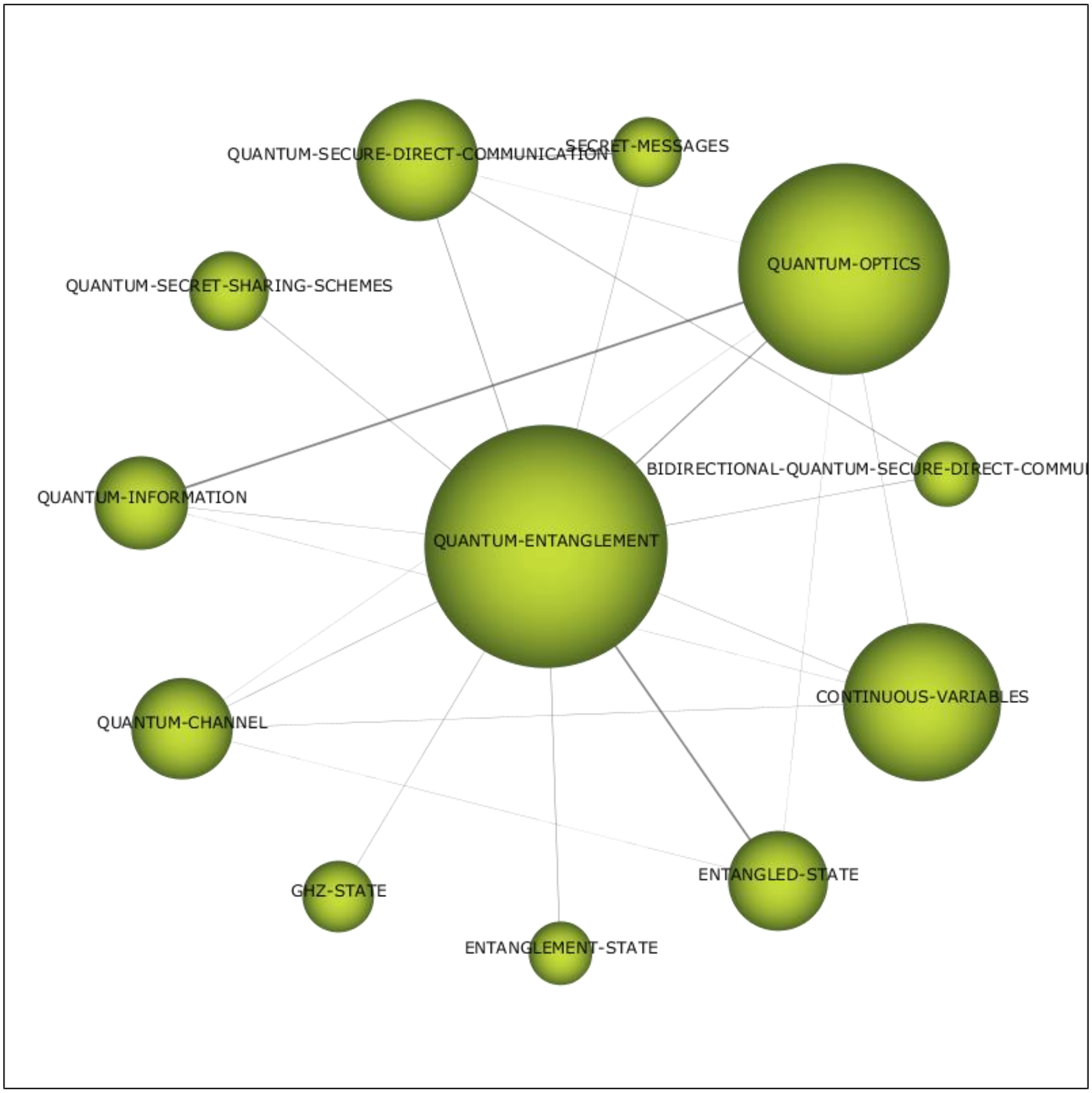

PUBLIC-KEY-CRYPTOGRAPHY

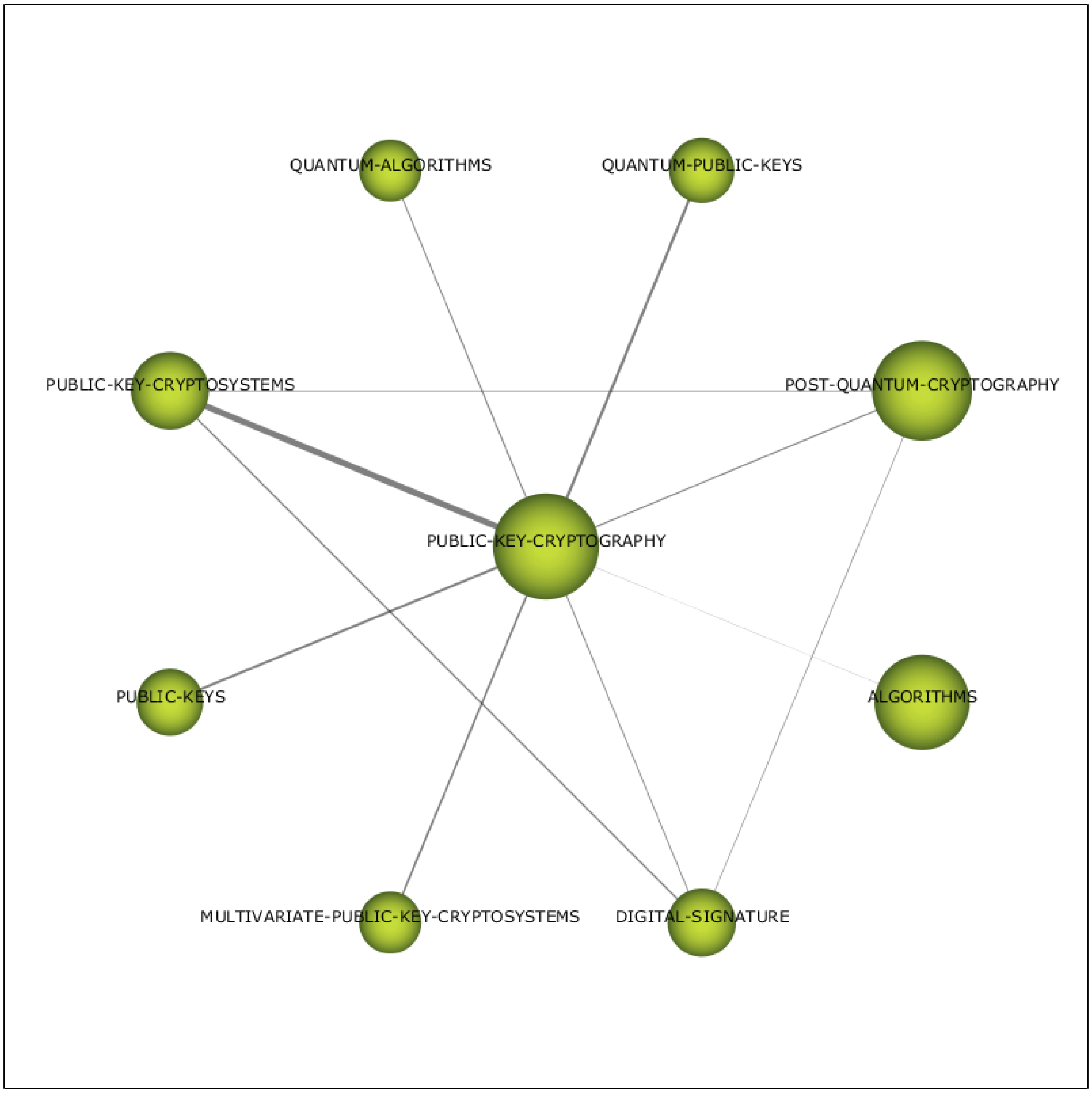

QUANTUM-SIGNATURE

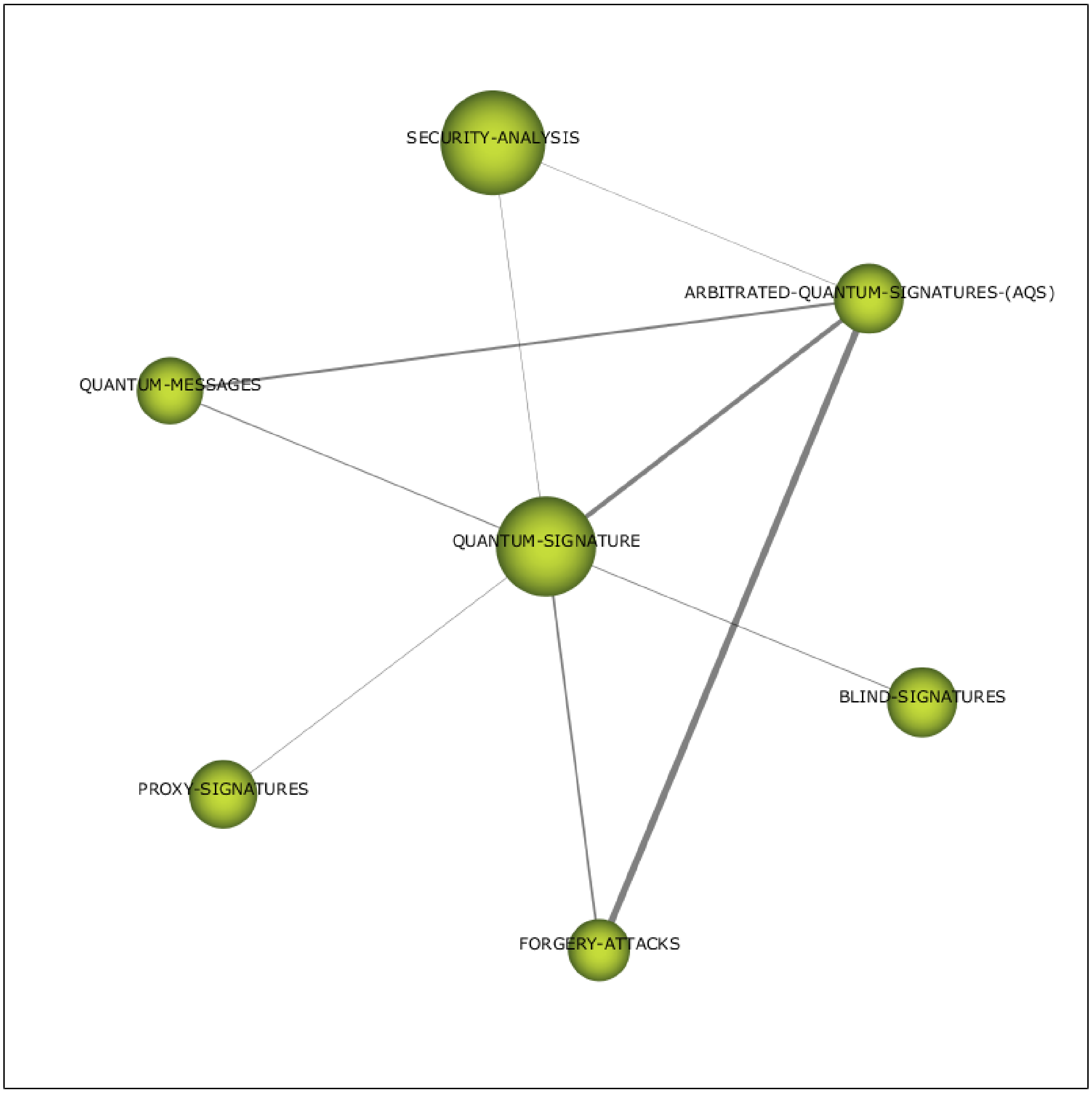

QUANTUM-KEY-DISTRIBUTION-SYSTEM

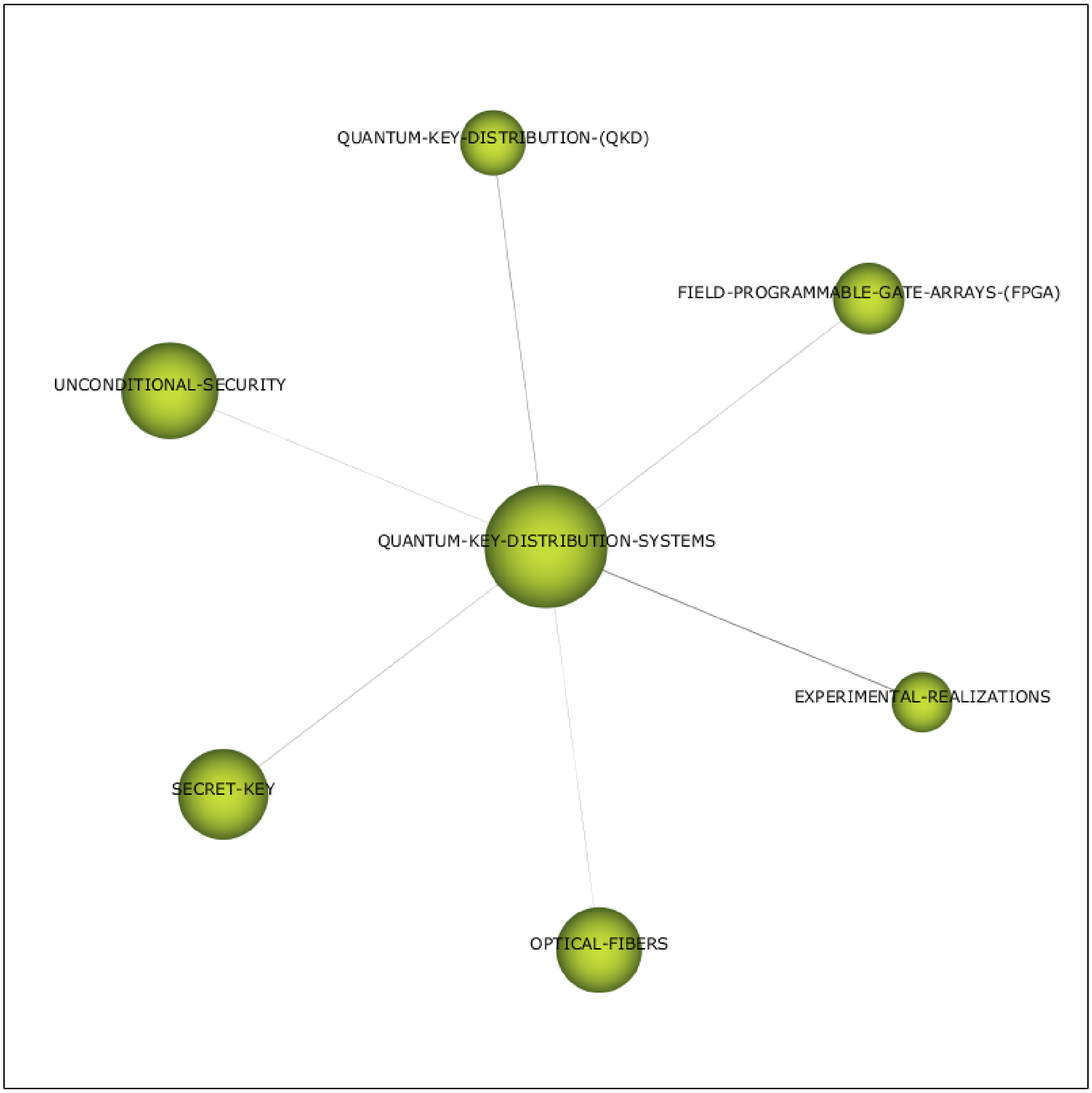

DECOY-STATE

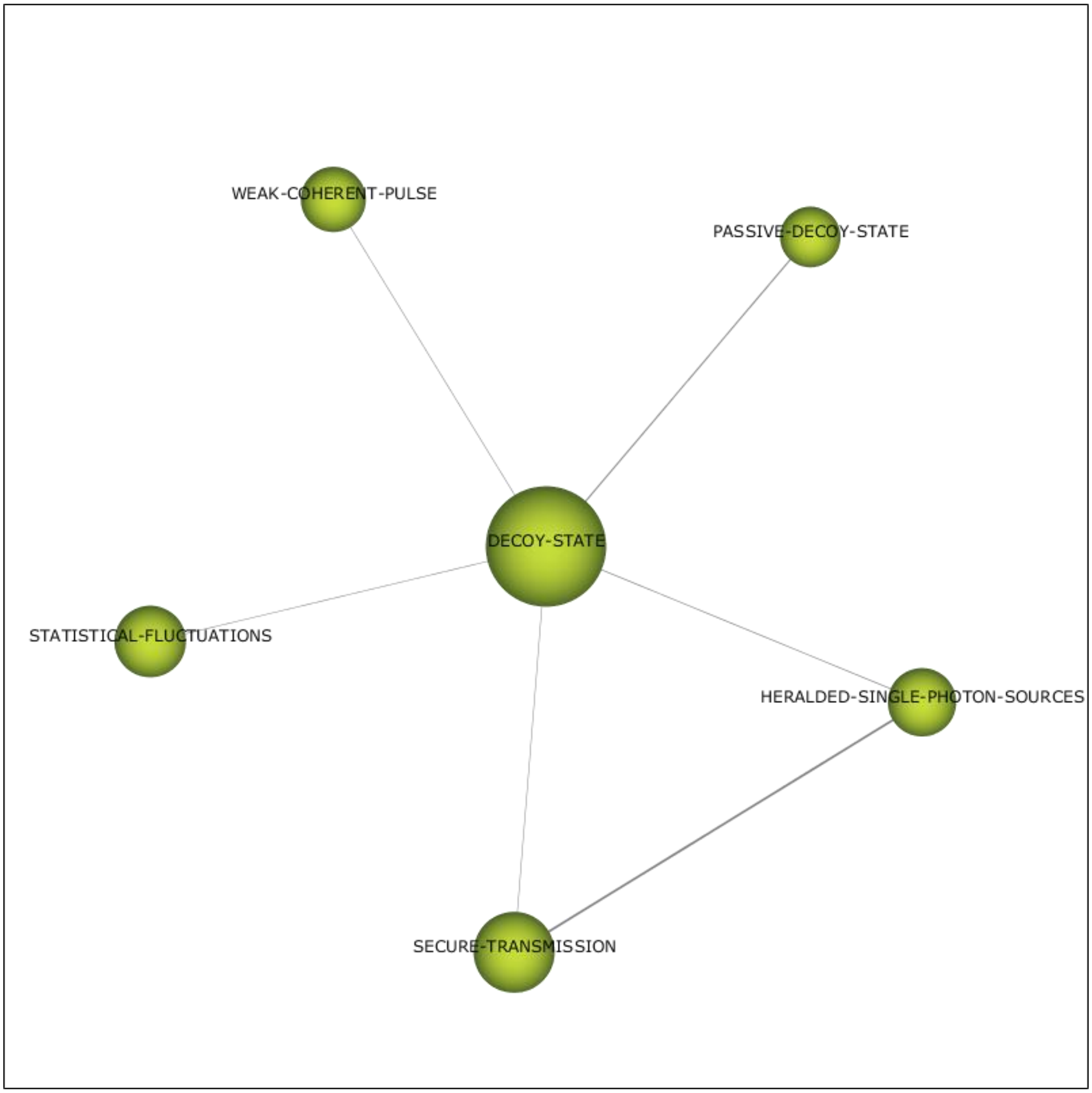
WEAK-COHERENT-PULSE
PASSIVE-DECOY-STATE
DECOY-STATE
STATISTICAL-FLUCTUATIONS
HERALDED-SINGLE-PHOTON-SOURCES
SECURE-TRANSMISSION

UNITARY-OPERATION

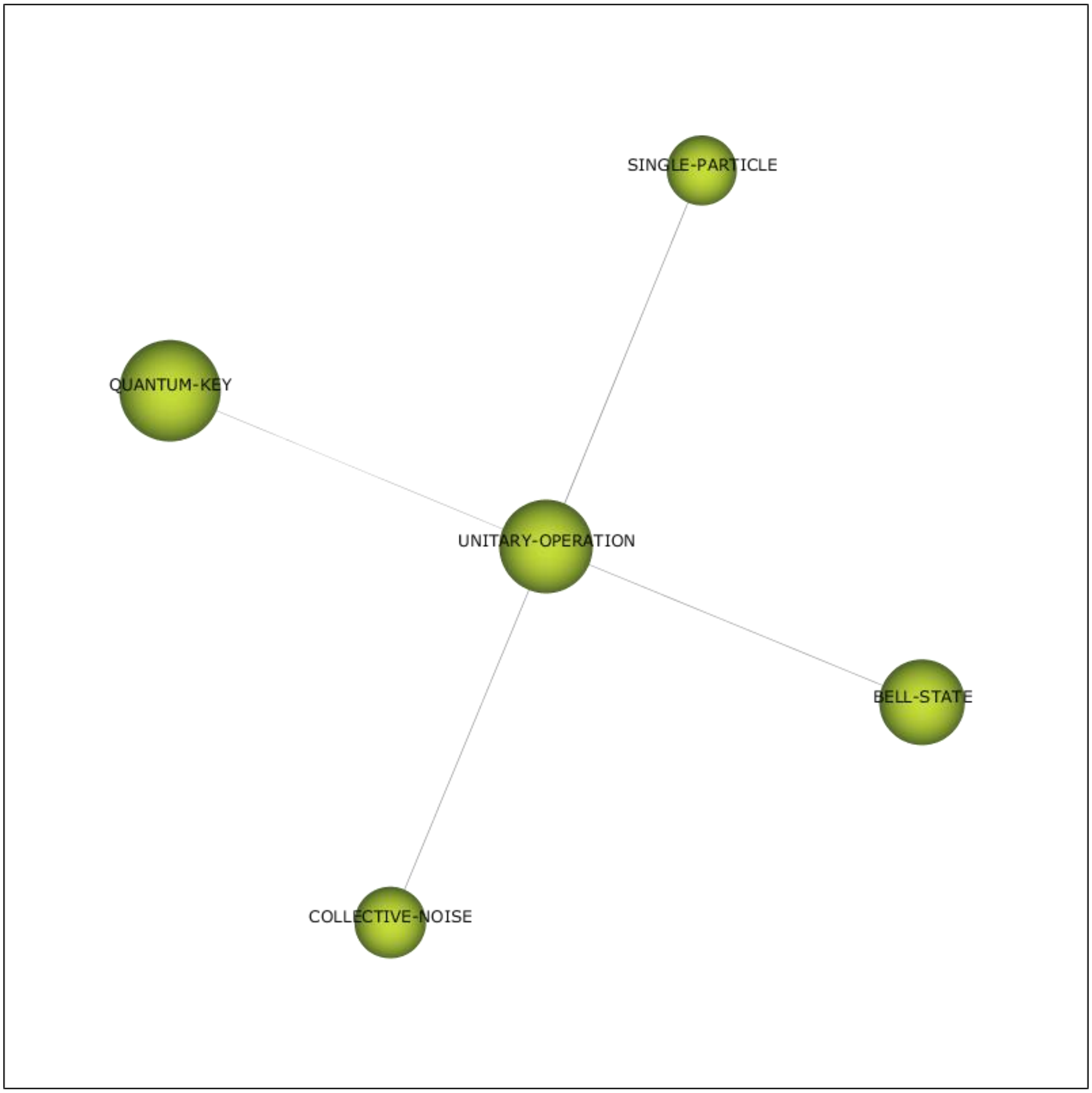

Supplement: S1 File — (PDF) [file pone.0190646.s001.pdf]
