# Supplementary material for: Examination of China’s performance and thematic evolution in quantum cryptography research using quantitative and computational techniques
Source: PLoS One. 2018 Jan 31;13(1):e0190646. doi: 10.1371/journal.pone.0190646 (PMC5791966; doi:10.1371/journal.pone.0190646)
Supplement: S2 File — (PDF) [file pone.0190646.s002.pdf]

S2 File. Calculating the Annual Average Growth Rate (AAGR) for performance measures.

$$AAGR = \frac{1}{N}\ln\left(\frac{P_N}{P_1}\right) * 100$$

Annual Average Growth Rate = (1/Number of transitions between periods)*ln(Amount in last period/Amount in first period)*100
