# Supplementary material for: Examination of China’s performance and thematic evolution in quantum cryptography research using quantitative and computational techniques
Source: PLoS One. 2018 Jan 31;13(1):e0190646. doi: 10.1371/journal.pone.0190646 (PMC5791966; doi:10.1371/journal.pone.0190646)
Supplement: S1 Table — (PDF) [file pone.0190646.s003.pdf]

S1 Table. Summarizing table with total performance measures from 2001-2017 along with their Annual Average Growth Rate (AAGR) from 2001-2016 for the top five quantum cryptography research countries.

**Countries/Territories**	**H-index (AAGR)**	**PF (AAGR)**	**CS (AAGR)**
US	232 (-11.9%)	1036 (6.9%)	30231 (12.8%)
CN	199 (1.0%)	1477 (19.8%)	11994 (36.5%)
UK	169 (-8.8%)	439 (3.4%)	18321 (10.4%)
DE	157 (-7.3%)	389 (7.3%)	12618 (20.2%)
CA	156 (7.3%)	436 (20.1%)	12515 (18.3%)
Median Totals	170 (-4.6)	498 (9.4%)	14311 (18.7%)
