# Supplementary material for: Examination of China’s performance and thematic evolution in quantum cryptography research using quantitative and computational techniques
Source: PLoS One. 2018 Jan 31;13(1):e0190646. doi: 10.1371/journal.pone.0190646 (PMC5791966; doi:10.1371/journal.pone.0190646)
Supplement: S2 Table — (PDF) [file pone.0190646.s004.pdf]

S2 Table. Publication Frequency table for top five quantum cryptography research countries from 2001-2017.

Year	CN	US	CA	UK	DE	Median
2001	10	32	2	21	9	10
2002	24	60	8	15	17	17
2003	16	63	9	12	25	16
2004	26	74	16	19	20	20
2005	36	86	21	28	24	28
2006	49	64	22	21	22	22
2007	62	47	21	17	16	21
2008	101	58	24	27	29	29
2009	99	51	34	32	36	36
2010	109	42	34	25	42	42
2011	110	54	41	39	44	44
2012	91	48	30	22	23	30
2013	150	74	41	36	20	41
2014	185	98	53	43	17	53
2015	179	86	34	43	18	43
2016	196	90	41	35	27	41
2017	34	9	5	4	0	5
Total	1477	1036	436	439	389	498
