# Supplementary material for: Examination of China’s performance and thematic evolution in quantum cryptography research using quantitative and computational techniques
Source: PLoS One. 2018 Jan 31;13(1):e0190646. doi: 10.1371/journal.pone.0190646 (PMC5791966; doi:10.1371/journal.pone.0190646)
Supplement: S3 Table — (PDF) [file pone.0190646.s005.pdf]

S3 Table. Citation Score table for top five quantum cryptography research countries from 2001-2017.

Year	CN	US	CA	UK	DE	Median
2001	8	431	115	407	62	115
2002	29	737	184	543	99	184
2003	76	818	166	513	192	192
2004	88	1095	231	565	271	271
2005	192	1628	394	817	462	462
2006	333	1720	490	996	558	558
2007	384	1914	507	995	657	657
2008	476	1872	527	999	595	595
2009	599	2248	691	1153	798	798
2010	735	2137	793	1196	903	903
2011	872	2206	861	1284	1134	1134
2012	1059	2200	937	1420	1153	1153
2013	1573	2450	1206	1538	1358	1538
2014	1847	2759	1658	1856	1516	1847
2015	1453	2569	1655	1768	1347	1655
2016	1900	2938	1784	1921	1289	1900
2017	370	509	316	349	224	349
Total	11994	30231	12515	18320	12618	14311
