# Supplementary material for: Examination of China’s performance and thematic evolution in quantum cryptography research using quantitative and computational techniques
Source: PLoS One. 2018 Jan 31;13(1):e0190646. doi: 10.1371/journal.pone.0190646 (PMC5791966; doi:10.1371/journal.pone.0190646)
Supplement: S4 Table — (PDF) [file pone.0190646.s006.pdf]

S4 Table. H-index table for top five quantum cryptography research countries from 2001-2017.

Year	CN	US	CA	UK	DE	Median
2001	6	18	1	15	6	6
2002	6	22	8	9	12	9
2003	6	20	9	12	10	10
2004	9	22	11	10	11	11
2005	11	24	12	14	15	14
2006	12	16	11	8	11	11
2007	12	11	8	10	8	10
2008	19	15	11	13	13	13
2009	19	16	13	12	16	16
2010	19	10	12	10	15	12
2011	21	11	17	12	14	14
2012	12	10	7	6	7	7
2013	17	14	13	13	7	13
2014	13	10	11	11	6	11
2015	9	9	9	9	4	9
2016	7	3	3	4	2	3
2017	1	1	0	1	0	1
Total	199	232	156	169	157	170
