# Supplementary material for: Examination of China’s performance and thematic evolution in quantum cryptography research using quantitative and computational techniques
Source: PLoS One. 2018 Jan 31;13(1):e0190646. doi: 10.1371/journal.pone.0190646 (PMC5791966; doi:10.1371/journal.pone.0190646)
Supplement: S5 Table — (PDF) [file pone.0190646.s007.pdf]

S5 Table. China's Collaboration Frequency for quantum cryptography research from 2001-2017.

Year	Collaboration Frequency
2001	2
2002	5
2003	10
2004	4
2005	6
2006	10
2007	3
2008	11
2009	10
2010	7
2011	14
2012	3
2013	43
2014	38
2015	29
2016	50
2017	13
